# m^6^A mRNA demethylase FTO regulates melanoma tumorigenicity and response to anti-PD-1 blockade

**DOI:** 10.1038/s41467-019-10669-0

**Published:** 2019-06-25

**Authors:** Seungwon Yang, Jiangbo Wei, Yan-Hong Cui, Gayoung Park, Palak Shah, Yu Deng, Andrew E. Aplin, Zhike Lu, Seungmin Hwang, Chuan He, Yu-Ying He

**Affiliations:** 1 Department of Medicine, Section of Dermatology, University of Chicago, Chicago, IL 60637 USA; 2 Departments of Chemistry, Department of Biochemistry and Molecular Biology, Institute for Biophysical Dynamics, University of Chicago, Chicago, IL 60637 USA; 3 Department of Pathology, University of Chicago, Chicago, IL 60637 USA; 4 Committee on Molecular Pathogenesis and Molecular Medicine, University of Chicago, Chicago, IL 60637 USA; 5 Department of Environmental Health, School of Public Health, China Medical University, Shenyang, Laoning, 110122 China; 6 Department of Cancer Biology, Thomas Jefferson University, Philadelphia, PA 19107 USA; 7 Sidney Kimmel Cancer Center, Thomas Jefferson University, Philadelphia, PA 19107 USA; 8 Howard Hughes Medical Institute, University of Chicago, Chicago, IL 60637 USA

**Keywords:** Cancer, Oncogenes, Skin cancer, Melanoma

## Abstract

Melanoma is one of the most deadly and therapy-resistant cancers. Here we show that N^6^-methyladenosine (m^6^A) mRNA demethylation by fat mass and obesity-associated protein (FTO) increases melanoma growth and decreases response to anti-PD-1 blockade immunotherapy. FTO level is increased in human melanoma and enhances melanoma tumorigenesis in mice. FTO is induced by metabolic starvation stress through the autophagy and NF-κB pathway. Knockdown of FTO increases m^6^A methylation in the critical protumorigenic melanoma cell-intrinsic genes including PD-1 (PDCD1), CXCR4, and SOX10, leading to increased RNA decay through the m^6^A reader YTHDF2. Knockdown of FTO sensitizes melanoma cells to interferon gamma (IFNγ) and sensitizes melanoma to anti-PD-1 treatment in mice, depending on adaptive immunity. Our findings demonstrate a crucial role of FTO as an m^6^A demethylase in promoting melanoma tumorigenesis and anti-PD-1 resistance, and suggest that the combination of FTO inhibition with anti-PD-1 blockade may reduce the resistance to immunotherapy in melanoma.

## Abstract (web summary)

FTO is an m6A demethylase. Here, the authors show that FTO promotes melanoma tumorigenicity and contributes to resistance to anti-PD1 blockade, while FTO inhibition sensitizes melanoma to anti-PD1 blockade.

## Introduction

Melanoma is one of the most deadly and therapy-resistant human cancers in the United States^1^. The incidence of melanoma continues to rise at an alarming rate each year, faster than any of the other common cancers^2^. In the past decades, tremendous progress has been made in elucidating the mechanism of melanoma development at the molecular, cellular and organismal levels^1^. Both genetic, including mutations in oncogenes and tumor suppressor genes, and epigenetic mechanisms, including DNA methylation, microRNAs, and other non-coding RNAs, have been demonstrated to play critical roles in melanoma pathogenesis^1,3–5^. In particular, the discoveries of BRAF mutations and their functions in most melanomas have led to the development of molecular therapy targeting the mutant BRAF^1^. However, the majority of the patients treated with an inhibitor of mutant BRAF eventually suffer continuous disease progression^6^. Recent breakthroughs in immunotherapy, including anti-PD-1 checkpoint blockade therapy, have benefitted a growing number of melanoma patients^7–9^. Still, more than half of these patients do not show a durable response to immunotherapy. Multiple mechanisms, such as driver mutations, epigenetic mechanisms, tumor plasticity, and immunosuppression, may cooperate to maintain the melanoma phenotype and mediate resistance to therapies, including immunotherapy^10,11^, which makes targeting a single pathway less effective. Our understanding of the molecular mechanisms for melanoma development and therapeutic response is still limited.

An emerging molecular mechanism regulating gene expression at the post-transcription level is N^6^-methyladenosine (m^6^A) RNA methylation. m^6^A RNA methylation is the most prominent chemical modification found in messenger RNA (mRNA) and non-coding RNA in eukaryotic cells^12–15^. Recently, the first m^6^A demethylase FTO was discovered, suggesting that m^6^A RNA methylation is reversible and dynamic and may have crucial physiological and pathological functions^16^. Subsequently, two studies independently mapped the transcriptome-wide m^6^A distribution^17,18^. These studies also suggest that, similar to the reversible epigenetic modifications to DNA and histone, post-transcriptional methylation of adenosines in RNA provides an epitranscriptomic layer of regulation that may control mRNA fate and gene expression.

In the past several years, compelling evidence has demonstrated that m^6^A methylation plays critical roles in controlling RNA metabolism and function in physiological processes and stress response. m^6^A modification regulates development, stem cell homeostasis, response to stresses such as heat-shock and genotoxic damage, and control of the circadian clock^19–22^. m^6^A modification regulates RNA fate and such functions as mRNA stability, nuclear processing, transport, localization, translation, primary microRNA processing, and RNA-protein interactions^23–28^. m^6^A modulators have been shown to play important roles in several cancers by regulating cancer-type-specific target genes and functions^19,20^, suggesting the unique regulatory and functional significance of the intrinsic m^6^A mRNA modifications and their regulators in different cancers. Recent work has also suggested that the genetic variations of FTO are associated with increased melanoma risk in humans^29,30^. The association of these FTO variations in exon 8 with melanoma risk seems to be independent of body mass index (BMI)^29,30^. As the first m^6^A demethylase identified, FTO has been show to act as an oncogenic factor in leukemia^31,32^ and glioblastoma^33^. However, the role of FTO as an m^6^A eraser in melanoma pathogenesis and response to anti-melanoma therapies remains poorly understood.

Here we report that the m^6^A demethylase FTO regulates melanoma growth and mediates melanoma resistance to anti-PD-1 antibody in vitro and in vivo. Our results identify a unique FTO-mediated and m^6^A-mediated mechanism in promoting melanoma tumorigenesis and resistance to anti-PD-1 blockade, and suggest that the combination of FTO inhibition with anti-PD-1 blockade may reduce resistance and improve the anti-melanoma response.

## Results

### FTO as a protumorigenic factor in melanoma

**Fig. 1:**
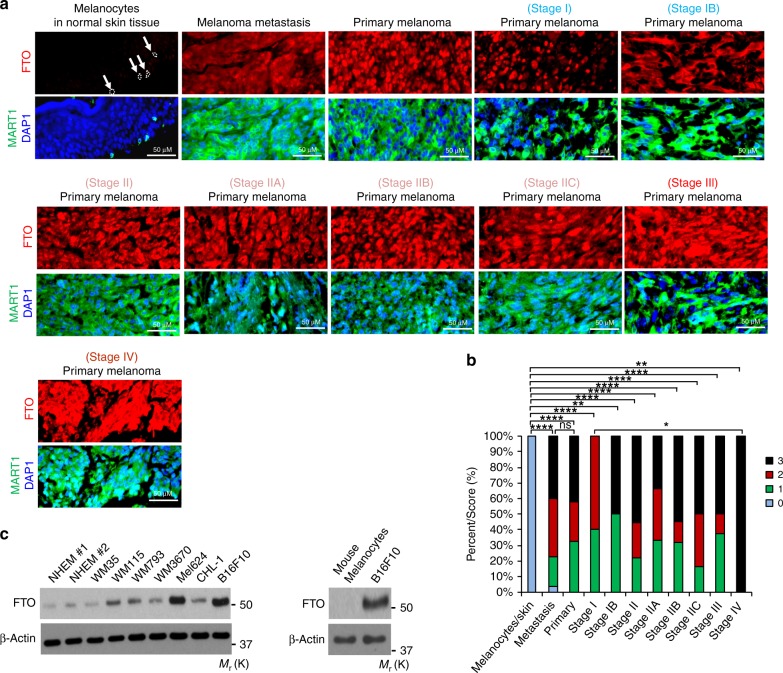
FTO is upregulated in human melanoma. **a** Immunofluorescence staining of FTO and MART1 in normal human skin and melanoma tissues. Nuclei are counterstained with DAPI in blue. Scale bar, 50 μm. The arrows indicate representative melanocytes in normal human epidermis. **b** Percentage of tumors (in stacked column format) for each score of FTO. 0 (Negative), 1 (Weak), 2 (Medium), and 3 (Strong); *P < 0.05; **P < 0.01; ****P < 0.0001; ns, *P* > 0.05, not significant; Mann–Whitney *U-*test. **c** Immunoblot analysis of the protein levels of FTO, and β-actin (loading control) in normal melanocytes and melanoma cells

To determine the role of FTO in human melanoma, we first analyzed FTO protein levels specifically in the melanocytes of normal human skin and malignant melanoma samples using immunofluorescence. We used MART1 immunofluorescence co-staining (Green) to specifically identify melanocytes in normal skin and melanoma cells (Fig. 1a). FTO (Red) was low in epidermal melanocytes in normal skin (*n* = 16), while it was significantly increased in all diagnoses of melanoma, including metastasis (*n* = 64), primary melanoma (*n* = 36), and melanoma at stages I–IV (*n* = 65) (Fig. 1a, b). There was no significant difference in FTO levels between different diagnosis groups of melanomas except between Stage I and IV. Next, we determined the FTO protein levels in normal human epidermal melanocytes (NHEM) and a panel of melanoma cell lines. FTO remained unchanged in WM35 (BRAF mutation, Human) cells (Fig. 1c). However, it was up-regulated in six of the tested cell lines, particularly in Mel624 (BRAF mutation, Human) and B16F10 (BRAF wild-type, Mouse) cells, two aggressive model melanoma cell lines, as well as WM115 (BRAF mutation, Human), WM793 (BRAF mutation, Human), WM3670 (BRAF and NRAS mutation, Human), and CHL-1 (BRAF and NRAS wild-type, Human) (Fig. 1c). These results demonstrate that FTO is upregulated in human melanoma samples and multiple melanoma cell lines, suggesting a protumorigenic role of FTO in melanoma development.

**Fig. 2:**
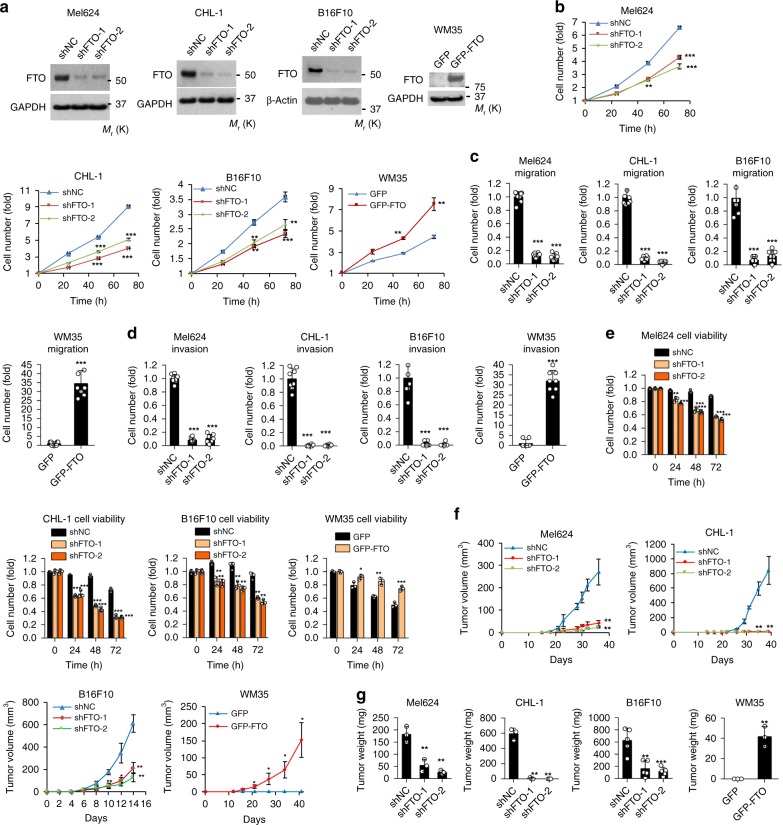
Effect of FTO knockdown or forced FTO expression in melanoma cells. **a** Confirmation of knockdown or forced expression of FTO in Mel624, CHL-1, B16F10, and WM35 by immunoblot analysis. **b** Cell proliferation assay in Mel624, CHL-1, B16F10, and WM35 with shNC (negative control), shFTO (FTO knockdown), GFP (vector control), or GFP-FTO (FTO overexpression). **c** Cell migration assay with cells as in **b**. **d** Cell invasion assay with cells as in **b**. **e** Cell viability assay in cells as in **b** but placed in suspension. **f** Average tumor volume (mm^3^) of Mel624, CHL-1, B16F10, and WM cells as in **b** at different days after subcutaneous injection in nude mice or C57BL/6 mice (*n* = 3). **g** Final tumor weight from F (*n* = 3). Data are shown as mean ± S.D. (*n* ≥ 3). *P < 0.05; **P < 0.01; ***P < 0.001; Student’s *t*-test

To determine the biological function of FTO in melanoma cells, we performed both gain- and loss-of-function studies in melanoma cells (Fig. 2a). Knockdown of FTO in FTO-high Mel624, CHL-1, and B16F10 cells decreased cell growth/proliferation, migration, invasion, and cell viability in cells placed in suspension (Fig. 2b–e, and Supplementary Fig. 1a–n). In addition, FTO knockdown also decreased cell invasiveness in 3D culture with Matrigel and colony formation in soft agar, respectively (S1O-S1S). Overexpression of FTO markedly increased cell proliferation, cell migration, invasion, and cell viability in cells in suspension (Fig. 2b–e, and Supplementary Fig. 1d) in WM35 with an FTO protein level similar to NHEM (Fig. 1c). To elucidate the role of FTO in melanoma pathogenesis in vivo, we performed xenograft and syngeneic melanoma tumorigenesis assays. Knockdown of FTO markedly reduced tumor volume and tumor growth in both Mel624 and CHL-1 cells in immunocompromised nude mice, and in B16F10 cells in immunocompetent C57BL/6 mice (Fig. 2f, g, and Supplementary Fig. 2a–j). Furthermore, forced overexpression of FTO in WM35 induced tumor growth in immunocompromised nude mice (Fig. 2f, g, and Supplementary Fig. 2d). These findings suggest that FTO is required for the malignant traits of melanoma cells in vitro and plays a protumorigenic role in melanoma tumor growth in vivo.

### Role of m^6^A in FTO function in melanoma cells

**Fig. 3:**
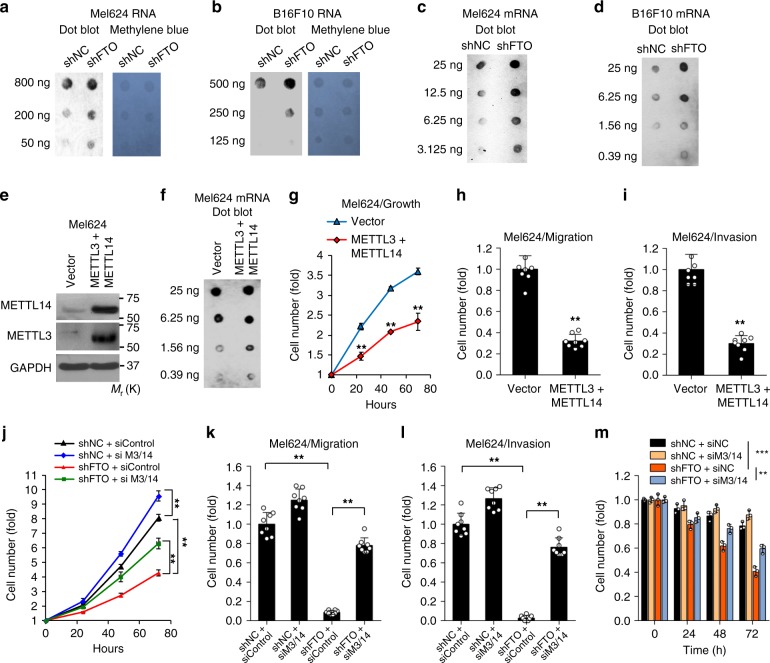
m^6^A enrichment in mRNA, and the function of m^6^A in melanoma cells. **a**, **b** m^6^A dot blot assays using total RNA of Mel624 and B16F10 cells with or without FTO knockdown. Methylene blue staining was used as a loading control. **c**, **d** m^6^A dot blot assays using poly(A) + mRNA of Mel624 and B16F10 cells with or without FTO knockdown. **e** Immunoblot analysis confirming forced overexpression of METTL3 and METTL14 in Mel624 cells. **f** m^6^A dot blot assays using poly(A) + mRNA in Mel624 with or without forced overexpression of METTL3 and METTL14. **g** Cell proliferation assay in Mel624 cells with or without forced overexpression of METTL3 and METTL14. **h** Cell migration assay in cells in G. **i** Cell invasion assay in cells in **g**. **j**–**m** Cell proliferation (**j**), migration (**k**), invasion (**l**), and cell viability (**m**) assays in suspension in shNC and shFTO of Mel624 with or without knockdown of METTL3 (M3) and METTL14 (M14). Data are shown as mean ± S.D. (*n* ≥ 3). *P < 0.05; **P < 0.01; ***P < 0.001; Student’s *t*-test

To examine the role of FTO in m^6^A regulation, we investigated the effect of FTO knockdown on m^6^A levels in mRNA^16,34^. Knockdown of FTO increased m^6^A levels in both total RNA and purified mRNA in both Mel624 and B16F10 cells (Fig. 3a–d). To determine the role of m^6^A in melanoma cell function, we performed gain-of-function tests of the m^6^A writers METTL3 (Methyltransferase Like 3) and METTL14 (Methyltransferase Like 14). Forced overexpression of METTL3 and METTL14 increased the total m^6^A level and decreased cell growth/proliferation (Fig. 3e–g), migration, and invasion (Fig. 3h, i, and Supplementary Fig. 3a, b). Furthermore, knockdown of METTL3 and METTL14 prevented the effect of FTO knockdown on cell growth/proliferation, migration, invasion, and cell viability in melanoma cells in suspension (Fig. 3j–m, and Supplementary Figs. 3e–j, 4a–f). The opposite was also true. Overexpression of METTL3 and METTL14 prevented the effect of FTO overexpression on these malignant traits (Supplementary Fig. 4g–l). These findings indicate that m^6^A modifications in RNA reduce melanoma cell proliferation and cell viability, suggesting a tumor-suppressive role of m^6^A in melanoma.

### Identification of potential target genes of FTO in melanoma

**Fig. 4:**
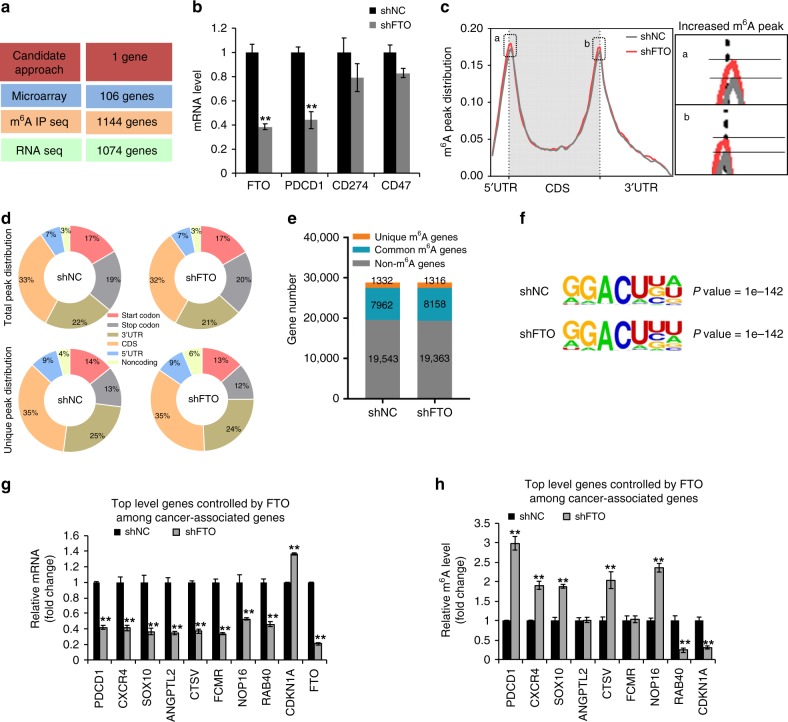
Identification of potential targets of FTO in melanoma. **a** Schematic summary for analysis of FTO target genes and number of genes identified. **b** Relative expression of FTO, PD-1 (PDCD1), CD274, and CD47 in Mel624 cells with or without FTO knockdown. **c** Distribution of m^6^A peaks across the length of mRNA. Region of 5-untranslated region (5′UTR) was binned into 10 segments, coding region (CDS) was binned into 50 segments, and 3-untranslated region (3′UTR) was binned into 40 segments, and the percentage of m^6^A peaks that fall within each bin was determined. **d** The proportion of m^6^A peak distribution in the indicated regions across the entire set of mRNA transcripts (top) and the appearance of new m^6^A peaks (unique peaks in shFTO), or loss of existing m^6^A peaks (unique peaks in shNC) after FTO knockdown (bottom). **e** Number of m^6^A-modified genes identified in m^6^A-seq in shNC and shFTO Mel624 cells. Common m^6^A genes contain at least one common m^6^A peak, while unique m^6^A genes contain no common m^6^A peaks. **f** Top consensus m^6^A motif identified by HOMER with m^6^A peaks in Mel624 cells with or without FTO knockdown. **g** qPCR analysis of mRNA levels of genes in Mel624 cells with shNC or shFTO. **h** Gene-specific m^6^A qPCR analysis of gene-specific m^6^A enrichment in Mel624 cells with shNC or shFTO. Data are shown as mean ± S.E. (*n* ≥ 3). *P < 0.05; **P < 0.01; ***P < 0.001; Student’s *t*-test

We next used both candidate and unbiased screening approaches to determine the potential mRNA targets of FTO in its melanoma-promoting function (Fig. 4a). First, we started with a candidate approach, focusing on the genes that are known to be associated with melanoma growth and in particular response to immunotherapy, including known melanoma-promoting genes PD-1 (PDCD1)^35^, PD-L1 (CD274)^11,36^, and CD47^37–39^. All three genes were expressed in Mel624 cells. We found that serum starvation induced the expression of FTO, PD-1 (PDCD1), and CD47, but not PD-L1 (CD274) (Supplementary Fig. 5a). FTO knockdown significantly decreased PD-1 (PDCD1) expression, but had no effect on PD-L1 (CD274) or CD47 (Fig. 4b). These data suggested that PD-1 is a potential FTO target gene in melanoma. Second, we performed microarray analysis of gene expression in MEL624 cells with or without FTO knockdown to determine transcriptome-wide changes upon FTO knockdown. We identified 106 genes that were significantly altered by FTO knockdown (Fig. 4a). Third, we used m^6^A- seq in combinaton with RNA-seq to identify more than 1000 potential transcriptome-wide m^6^A-modifiedgene targets for FTO (Fig. 4a), including the melanoma-promoting genes CXCR4 and SOX10. m^6^A peaks detected in poly(A)+-enriched RNAs showed reproducible patterns of methylation (Supplementary Fig. 5b). FTO knockdown increased m^6^A enrichment in 5′UTR and 3′UTR, while it had little effect on the CDS region (Fig. 4c), and altered total peak distribution and unique peak distribution (Fig. 4d). FTO knockdown cells also showed an increased number of common m^6^A genes (Fig. 4e) and fewer m^6^A peaks (Supplementary Fig. 5c), likely due to the degradation of m^6^A-modified mRNAs. Sequence analysis of m^6^A peaks showed the previously identified m^6^A target sites (GGACU)^17^ (Fig. 4f). However, PD-1 (PDCD-1) was undetectable in either microarray or RNA-seq analysis (Supplementary Fig. 5d), possibly due to the limitations of these assays. m^6^A IP seq analysis showed that FTO knockdown increased the m^6^A levels in the transcripts of CXCR4 and SXO10 (Supplementary Fig. 5e, f). Next, we validated a set of downregulated genes (PD-1 (PDCD1), CXCR4, SOX10, ANGPTL2, CTSV, FCMR, NOP16, and RAB40), and upregulated genes (CDKN1A) upon FTO knockdown (Fig. 4g, and Supplementary Fig. 5g–i). Consistently, m^6^A IP qPCR analysis showed that FTO knockdown increased the m^6^A levels in the transcripts of PD-1 (PDCD1), CXCR4, SOX10, CTSV, and NOP16 (Fig. 4h), suggesting these genes as potential targets of FTO.

### Identification of functionally critical target genes of FTO

**Fig. 5:**
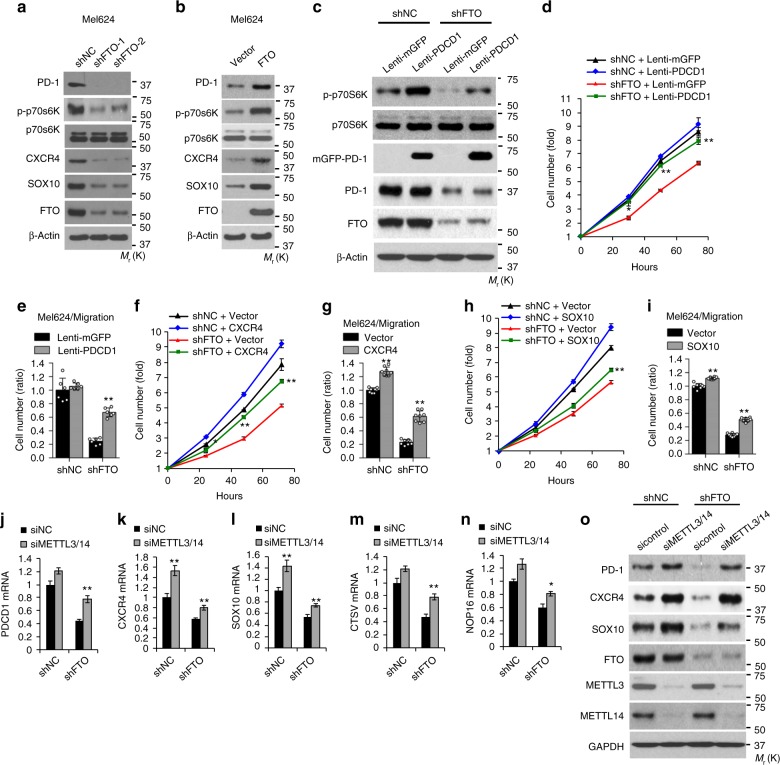
PD-1 (PDCD1), CXCR4, and SOX10 are critical target genes of FTO in melanoma cells. **a**, **b** Immunoblot analysis of PD-1, p-p70S6K, p70S6K, CXCR4, SOX10, FTO, and β-actin in Mel624 stable cells with or without knockdown (**a**) and forced overexpression (**b**) of FTO. **c** Immunoblot analysis of PD-1, p-p70S6K, p70S6K, FTO, and β-actin in Mel624 with or without FTO knockdown, and/or PD-1 (PDCD1) overexpression. **d** Cell proliferation assay in cells as in **c**. **e** Cell migration assay in cells as in **c**. **f**, **g** Cell proliferation (**f**) and migration analysis (**g**) in Mel624 with or without FTO knockdown, and/or CXCR4 overexpression. **h**, **i** Cell proliferation (**h**) and migration analysis (**i**) in Mel624 with or without FTO knockdown, and/or SOX10 overexpression. **j**–**n** qPCR analysis of the expression of PD-1 (PDCD1) (**j**), CXCR4 (**k**), SOX10 (**l**), CTSV (**m**), and NOP16 (**n**) in Mel624 cells with or without FTO knockdown, in combination with siRNA knockdown of both METTL3 and METTL14. **o** Immunoblot analysis of PD-1, CXCR4, SOX10, FTO, METTL3, METTL14, and GAPDH in cells as in **j**–**n**. Data are shown as mean ± S.D. (**d**–**i**), or mean ± S.E. (**j**–**n**) (*n* ≥ 3). *P < 0.05; **P < 0.01; ***P < 0.001; Student’s *t*-test

Among the genes identified above, PD-1 has been shown to be expressed in some melanoma cells and act as an intrinsic protumorigenic factor to promote melanoma tumor growth^35^. It activates the downstream of mTOR signaling^35^, demonstrating a critical intrinsic role of PD-1 in melanoma. In addition, PD-1 has been successfully targeted in immune cells for cancer immunotherapy^36^. Therefore understanding the regulation of PD-1 may have a broader impact on cancer biology and immunotherapy response in melanoma. In addition, CXCR4 and SOX10 are critical melanoma-promoting genes^40–42^. Thus we decided to focus on investigating whether PD-1, CXCR4, and SOX10 act as crucial targets of FTO. Additional shRNAs targeting FTO confirmed that FTO regulates the expression of PD-1 (PDCD1), CXCR4, and SOX10 (Fig. 5a, and Supplementary Fig. 6a–c). Knockdown of FTO reduced the protein levels of PD-1, CXCR4, and SOX10, as well as the phosphorylation of p70s6K, a substrate of mTOR activation (Fig. 5a, and Supplementary Fig. 6a–c). The opposite was also true. Forced overexpression of FTO increased the protein and mRNA levels of these genes as well as the phosphorylation of p70s6K (Fig. 5b, and Supplementary Fig. 6d). Furthermore, forced overexpression of wild-type (WT) FTO increased the mRNA and protein levels of PD-1 (PDCD1), CXCR4, and SOX10, and cell proliferation, whereas demethylase-inactive mutants^16^ had no effect (Supplementary Fig. 6e–h). These findings indicate that FTO is responsible for promoting the expression of melanoma-intrinsic PD-1, CXCR4, and SOX10.

Next we investigated whether these melanoma-promoting genes are responsible for FTO’s function. Forced overexpression of PD-1 (PDCD1) in FTO knockdown cells increased phosphorylation of p70s6K (Fig. 5c), accompanied by significantly increased cell growth/proliferation (Fig. 5d) and migration (Fig. 5e, and Supplementary Fig. 7a). Similarly, forced overexpression of CXCR4 and SOX10 in FTO knockdown cells significantly increased cell growth/proliferation and migration (Fig. 5f–i, and Supplementary Fig. 7b–e). These results demonstrate that PD-1, CXCR4, and SOX10 are critical downstream targets of FTO responsible for its function in melanoma. Indeed, all melanoma cell lines showed increased PD-1 protein levels as compared with normal melanocytes (Supplementary Fig. 7f). PD-1 (PDCD1) mRNA expression is positively associated with FTO expression in melanoma (Supplementary Fig. 7g, h). In addition, we found a positive association between FTO and PD-1 protein levels in Mel624 cells (Supplementary Fig. 7i, j) and human melanoma tissue (Supplementary Fig. 7k–m).

To determine whether regulation of these melanoma-promoting genes by FTO is m^6^A-dependent, we assessed the role of m^6^A by knocking down the m^6^A methyltransferases METTL3 and METTL14 (Supplementary Fig. 8a–c). In FTO knockdown cells, subsequent knockdown of both METTL3 and METTL14 significantly increased the mRNA levels of PD-1 (PDCD1), CXCR4, SOX10, CTSV2, and NOP16 (Fig. 5j–n, and Supplementary Figs. 8a–d, 9a, b), and the protein levels of PD-1, CXCR4, and SOX10 (Fig. 5o), respectively, to a level similar to that in the control cells (Fig. 5j–o). These findings demonstrate that FTO regulates the expression of its target genes via m^6^A RNA modification and its demethylase activity.

### Role of YTHDF2 in FTO’s function in melanoma

**Fig. 6:**
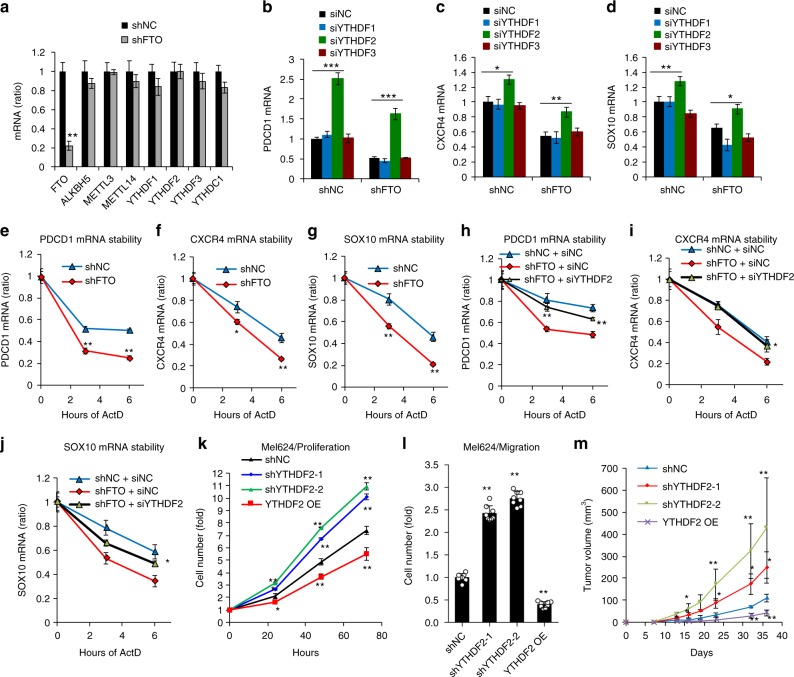
FTO regulates its target gene expression through suppressing m^6^A/YTHDF2-mediated mRNA decay. **a** qPCR analysis of mRNA levels in Mel624 stable cells of shNC or shFTO. **b**–**d** qPCR analysis of the mRNA levels of PD-1 (PDCD1) (**b**), CXCR4 (**c**), and SOX10 (**d**) in Mel624 cells with or without FTO knockdown, in combination with siRNA knockdown of the m^6^A readers YTHDF1-3. **e**–**g** qPCR analysis of the mRNA stability of PD-1 (PDCD1) (**e**), CXCR4 (**f**), and SOX10 (**g**) in Mel624 cells with or without FTO knockdown. **h-j** qPCR analysis of the mRNA stability of PD-1 (PDCD1) (**h**), CXCR4 (**i**), and SOX10 (**j**) in Mel624 cells with or without FTO knockdown in combination with or without siRNA knockdown of YTHDF2. **k**, **l** Cell proliferation (**k**) and migration (**l**) analysis in Mel624 with or without knockdown or forced overexpression of YTHDF2. **m** Tumor growth of Mel624 cells with or without knockdown or forced overexpression of YTHDF2 after subcutaneous injection in nude mice (*n* = 3). Data are shown as mean ± S.E. (**a**–**k**), or mean ± S.D. (**l** and **m**) (*n* ≥ 3). *P < 0.05; **P < 0.01; Student’s *t*-test

The function of m^6^A in regulating gene expression is executed mostly through the readers, including YTH domain-containing family proteins in mammalian cells^17^. FTO knockdown did not affect the expression of critical m^6^A regulators (Fig. 6a). To determine how m^6^A RNA methylation regulates gene expression, we analyzed the role of YTHDF1-3. Knockdown of YTHDF1 or YTHDF3 had no effect on the mRNA levels of PD-1 (PDCD1), CXCR4, or SOX10 in control and FTO-knockdown cells. However, knockdown of YTHDF2 significantly increased the mRNA levels of all three genes in both control and FTO-knockdown cells (Fig. 6b–d, and Supplementary Fig. 10a–c). Moreover, knockdown of FTO decreased the mRNA stability of PD-1 (PDCD1), CXCR4, and SOX10 (Fig. 6e–g). Knockdown of YTHDF2 increased the mRNA stability of PD-1 (PDCD1), CXCR4, and SOX10 in shFTO cells (Fig. 6h–j). Consistently, knockdown of YTHDF2 increased, while forced overexpression of YTHDF2 decreased, cell proliferation and migration in melanoma cells in vitro (Fig. 6k, l, and Supplementary Fig. 10d–f) as well as tumor growth in vivo (Fig. 6m, and Supplementary Fig. 10g, h), supporting a tumor suppressor role for YTHDF2. These findings indicate that YTHDF2-mediated RNA decay controls the expression of the FTO target genes in melanoma cells.

### FTO expression is induced by metabolic stress

**Fig. 7:**
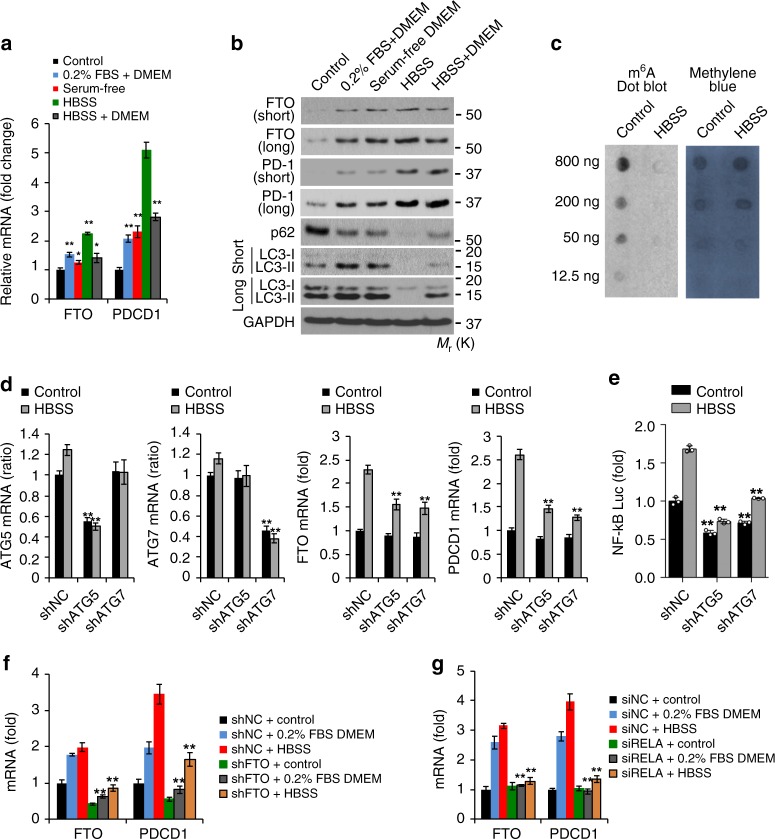
Metabolic stress induces FTO expression through NF-kB and autophagy. **a** qPCR analysis of FTO and PD-1 (PDCD1) mRNA levels in Mel624 cells cultured with control medium (10% FBS DMEM), 0.2% FBS DMEM, serum-free DMEM, Hanks’ balanced salt solution containing calcium and magnesium (HBSS), or a combination of DMEM and HBSS. **b** Immunoblot analysis of FTO, PD-1, p62, LC3-I/II (short and long exposure), and GAPDH in Mel624 cells treated as in **a**. **c** m^6^A dot blot assays using total RNA of Mel624 cultured with control medium or HBSS. Methylene blue staining was used as a loading control. **d** qPCR analysis of FTO and PD-1 (PDCD1) mRNA levels in Mel624 cells with or without knockdown of ATG5 or ATG7 and with or without metabolic stress. **e** Luciferase reporter analysis of NF-κB response element in Mel624 cells as in **d**. **f** qPCR analysis of FTO and PD-1 (PDCD1) mRNA levels in Mel624 cells with or without FTO knockdown treated with or without metabolic stress. **g** qPCR analysis of FTO and PD-1 (PDCD1) mRNA levels in Mel624 cells with or without siRNA knockdown of RELA treated as in **e**. Data are shown as mean ± S.E. (**a**, **d**, **f**, **g**), or mean ± S.D. (**e**) (*n* ≥ 3).*P < 0.05; **P < 0.01; ***P < 0.001; Student’s *t*-test

To investigate potential pathways by which FTO is upregulated in melanoma cells, we analyzed the role of metabolic stress, since cancer cells acquire adaptive capabilities to survive and grow under metabolic stress. We found that metabolic stress conditions, such as low serum (0.2% FBS) or model starvation medium (Hank’s Balanced Salt Solution, HBSS, lacking serum, amino acids and glucose) increased the expression of FTO and PD-1 (PDCD1) at the mRNA (Fig. 7a, and Supplementary Fig. 5a) and protein levels (Fig. 7b, and Supplementary Fig. 11a, b). Consistently, HBSS decreased the m^6^A level (Fig. 7c). Since these conditions are also known to induce autophagy (Fig. 7b, and Supplementary Fig. 11a, b), we reasoned that FTO induction by metabolic stress is mediated through the autophagy pathway. Indeed, knockdown of the autophagy essential genes ATG5 or ATG7 reduced starvation-induced FTO and PD-1 (PDCD1) expression, as well as NF-κB activity (Fig. 7d, e). Knockdown of FTO decreased PD-1 (PDCD1) expression (Fig. 7f). Knockdown of the NF-κB subunit p65 (RELA) markedly reduced the induction of FTO and PD-1 (PDCD1) by metabolic stress (Fig. 7g). These results demonstrate that FTO and PD-1 (PDCD1) are induced by metabolic stress through the autophagy and NF-κB pathways.

### Role of FTO on anti-PD-1 blockade and IFNγ

**Fig. 8:**
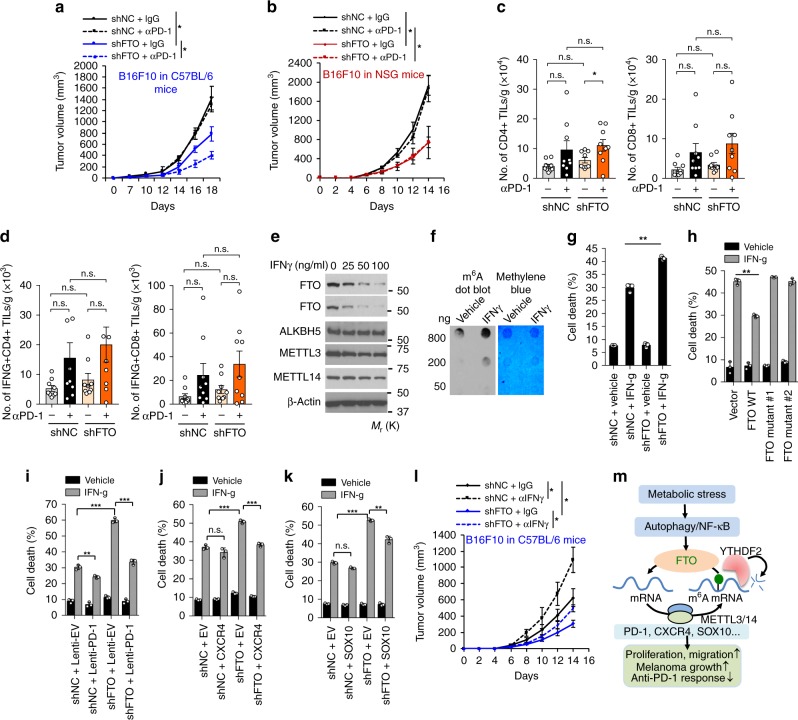
Role of FTO in melanoma cell response to anti-PD-1 antibody in vivo or to interferon gamma (IFNγ) in vitro. **a**, **b** Tumor growth kinetics of control or FTO-knockdown B16F10 cells in C57BL/6 mice (**a**) and NSG mice (**b**) treated with anti-PD-1 or isotype control antibody (*n* = 4–6). *P < 0.05. **c**, **d** Flow cytometric analysis of the number of CD4+ and CD8+ TILs (**c**) and IFNγ-producing TILs (**d**) per gram of tumor tissue (*n* = 9). Mann–Whitney *U-*test. *P < 0.05. n.s., not significant. **e** Immunoblot analysis of FTO, ALKBH5, METTL3, METTL14, and GAPDH in Mel624 cells treated with or without IFNγ (100 ng/ml) for 24 h. **f** m^6^A dot blot assays using total RNA of Mel624 cells treated with or without IFNγ (100 ng/ml) for 24 h. **g** Apoptosis assay in Mel624 cells with or without FTO knockdown and treatment with or without IFNγ (50 ng/ml) for 48 h. **h** Apoptosis assay in Mel624 cells overexpressing vector, FTO WT, mutant 1, or mutant 2, and treated with or without IFNγ (50 ng/ml) for 48 h (*n* = 3). **P < 0.01, Student’s *t*-test. **i**–**k** Apoptosis assay in Mel624 cells with or without FTO knockdown overexpressing vector or PD-1 (**i**), CXCR4 (**j**), or SOX10 (**k**) treated with or without IFNγ (50 ng/ml) for 48 h (*n* = 3). *P < 0.05; **P < 0.01; ***P < 0.001; Student’s *t*-test. **l** Tumor growth of control or FTO-knockdown B16F10 cells in C57BL/6 mice treated with the anti-IFNγ antibody or isotype control IgG (*n* = 4–6). Data are shown as mean ± S.E. (**a**–**e**, and **l**), or mean ± S.D. (**g**–**k**) (*n* ≥ 3). *P < 0.05; **P < 0.01; ***P < 0.001; Student’s *t*-test. **m** Proposed model of the regulatory and functional role for FTO in melanoma pathogenesis and response to anti-PD-1 blockade

Although immunotherapy has been effective in producing long-lasting therapeutic effects on several aggressive cancers in a subset of patients^7–9^, understanding the mechanism of resistance is critical to broaden the clinical application of immunotherapy for patients with advanced cancers^11^. Since FTO promotes melanoma tumorigenicity and regulates the expression of tumor-promoting melanoma cell-intrinsic PD-1 as well as CXCR4 and SOX10, we reasoned that FTO may also regulate the response of melanoma to immunotherapy. To determine whether FTO inhibition affects immunotherapy response, we treated C57BL/6 mice bearing B10F10 melanomas with anti-PD-1 antibody or isotype control IgG. Anti-PD-1 antibody had no effect on tumor growth of control B16F10 cells (Fig. 8a), consistent with previous studies^35,43^, mimicking resistance to PD-1 blockade in melanoma immunotherapy. However, anti-PD-1 antibody significantly inhibited tumor growth for FTO knockdown tumors (Fig. 8a). Next, we assessed whether the immune system is required for the effect of FTO knockdown on anti-PD-1 blockade. In immunodeficient NSG (severely combined immunodeficient (NOD/SCID) interleukin-2 receptor (IL-2R) gamma chain null) mice, anti-PD-1 antibody had no effect on tumor growth in either control or B16F10 cells with FTO knockdown (Fig. 8b). These results indicate that the effect of FTO knockdown on melanoma response to anti-PD-1 immunotherapy is dependent on the immune system.

To understand the mechanism by which FTO inhibition sensitizes melanoma to anti-PD-1 blockade, we first analyzed whether FTO knockdown affects T cell infiltration into tumors after PD-1 blockade (Supplementary Fig. 12a). The PD-1 blockade increased CD4^+^ tumor-infiltrating lymphocyte (TIL) numbers in B16F10 melanoma with FTO knockdown, but not in control cells (Fig. 8c). Notably, no significant difference was seen in CD8^+^ TIL numbers in either control or FTO-knockdown cells (Fig. 8c). We also assessed the IFNγ production of CD4^+^ and CD8^+^ TILs on day 14 after inoculation. However, FTO knockdown in melanoma cells did not affect the number of IFNγ-producing CD4^+^ or CD8^+^ TILs s (Fig. 8d). Although further investigation is necessary to establish the effect of FTO knockdown and anti-PD-1 treatment on other immune cells and other immune mediators, these data suggest that FTO knockdown did not affect the infiltration of IFNγ-producing cytotoxic TILs.

Next we analyzed the role of FTO in the response of melanoma cells to IFNγ. First, we assessed the effect of IFNγ on m^6^A regulators. IFNγ downregulated FTO in a dose-dependent manner, but had no effect on the protein levels of other regulators such as ALKBH5, METTL3, or METTL14 (Fig. 8e). Consistently, IFNγ also increased m^6^A levels (Fig. 8f), while it decreased PD-1 protein levels (Supplementary Fig. 12b). Next, we investigated whether FTO knockdown sensitizes melanoma cells to cell death induced by IFNγ. FTO knockdown did indeed sensitize melanoma cells to IFNγ-induced growth inhibition and cell killing (Fig. 8g, and Supplementary Fig. 12c). The converse was also true: Overexpression of WT FTO suppressed IFNγ-induced melanoma cell death, while overexpression of FTO mutants had no effect (Fig. 8h). These data suggest a critical role of FTO in the resistance of melanoma cells to IFNγ. Furthermore, we found that overexpression of PD-1 (PDCD1), CXCR4, or SOX10 inhibited IFNγ-induced cell death in FTO-knockdown melanoma cells (Fig. 8i–k). Consistently, blocking IFNγ by anti-IFNγ antibody increased tumor growth of both control and B16F10 melanoma cells with FTO knockdown in C57BL/6 mice (Fig. 8l). These results demonstrate that FTO inhibition enables a response to anti-PD-1 blockade in melanoma in vivo, and to IFNγ-induced killing in melanoma cells in vitro through regulating melanoma cell-intrinsic PD-1, CXCR4, and SOX10. Taken together, these results demonstrate a critical role of melanoma cell-intrinsic FTO in promotinging melanoma resistance to anti-PD-1 blockade, suggesting that FTO inhibition can reduce resistance to anti-PD-1 therapy (Fig. 8m).

## Discussion

Here we found that the obesity-associated protein FTO, also an m^6^A eraser, plays an important role in melanoma (Fig. 8m). FTO is increased in melanoma, suggesting a protumorigenic role of FTO in melanoma. FTO increases proliferation, migration, and invasion in melanoma cells in vitro and melanoma tumor growth in vivo. FTO can be upregulated by metabolic stress and starvation, a metabolic challenge that tumor cells are constantly facing in vivo, suggesting that the induction of FTO serves as an adaptive mechanism to metabolic stress in melanoma cells to promote proliferation, invasion, and migration. At the molecular level, FTO knockdown increases m^6^A enrichment in the 5′UTR and 3′UTR regions across the whole transcriptome. FTO knockdown increases m^6^A enrichment in the critical melanoma-promoting genes including PD-1 (PDCD1), CXCR4, and SOX10, and decreases their mRNA stability. The RNA decay of these genes in FTO knockdown cells is mediated by the m^6^A reader YTHDF2. YTHDF2 knockdown increases melanoma growth, whereas YTHDF2 overexpression decreases it, supporting a tumor suppressor role for YTHDF2 in melanoma. The regulation of these three genes by FTO is mediated through m^6^A demethylation and FTO’s demethylase activity. Lastly, FTO plays an important role in therapeutic resistance to anti-PD-1 immunotherapy and cell killing by IFNγ, a critical mechanism in immunotherapy. Overall, our findings demonstrate a crucial role of the m^6^A demethylase FTO in melanoma tumorigenesis and response to immunotherapy (Fig. 8m).

Our findings demonstrate that melanoma genes are regulated by FTO through m^6^A RNA methylation. FTO has been shown to be an RNA demethylase for m^6^A^16^ and also works on near cap m^6^A_m_^44^, with opposite effects on RNA stability. FTO-mediated m^6^A_m_ demethylation was reported to promote mRNA decay^44^ and thus FTO could decrease transcript levels with m^6^A_m_ demethylation. However, recent studies indicated that the effect of FTO on mRNA stability is mostly through m^6^A but not m^6^A_m_^45^. Importantly, the cap m^6^A_m_ methyltransferase PCIF1 was recently identified, and PCIF1 deletion eliminated cap m^6^A_m_ but had minimal effects on target mRNA stability^46^, inconsistent with the previous report^44^, confirming the minimal effect of cap m^6^A_m_ on mRNA stability. We found that the regulation of PD-1 (PDCD1), CXCR4, and SOX10 all appear to be partially mediated through the latter m^6^A/YTHDF2 mechanism, because: (1) FTO knockdown decreased the mRNA levels and stability of these melanoma genes, in parallel with increased m^6^A enrichments; (2) knockdown of the m^6^A reader YTHDF2 increased the mRNA levels and stability of these genes, reversing the effect of FTO knockdown; (3) knockdown of METTL3 and METTL14 reversed the effect of FTO knockdown; (4) forced overexpression of YTHDF2 phenocopies the effect of FTO knockdown in cell proliferation, migration, and tumor growth. These results suggest that m^6^A enrichment lead to RNA decay of these genes through a YTHDF2-dependent mechanism in melanoma. Nevertheless, it is still possible that FTO-regulated demethylation could have other additional effects that remain to be uncovered.

We found that FTO plays a crucial role in metabolic pathways in melanoma cells. First, FTO regulates PD-1 (PDCD1) expression, thus FTO-mediated demethylation promotes mTOR signaling and cell growth/proliferation. Previously, FTO has been shown to regulate nutrient sensing and mTOR^47^. Here we identified an epitranscriptomic mechanism by which FTO regulates mTOR signaling through m^6^A-mediated tuning of the PD-1 (PDCD1) gene. Second, we found that metabolic stress and starvation induces FTO expression in melanoma cells. This metabolic stress-induced FTO expression requires autophagy and NF-κB. Due to uncontrolled proliferation of tumor cells in vivo, tumor cells likely face constant metabolic stress and lack of sufficient nutrient supply. It is possible that FTO induced by starvation promotes melanoma cell proliferation and/or survival. Our findings suggest FTO as a critical adaptation mechanism in melanoma cells under metabolic stress.

Anti-PD-1 checkpoint blockade therapy has demonstrated an unprecedented anti-tumor response rate in advanced cancers, including melanoma^8,9^, and reduced immune-related adverse side effects compared to ipilimumab^48^. However, more than half of such patients do not show a durable response to immunotherapy^10^. Recent studies have shown that melanoma intrinsic PD-1 is one of the critical intrinsic tumor-promoting regulators in melanoma, even in mice lacking adaptive immunity^35^. However, it has been unclear how PD-1 expression is regulated in melanoma cells. We found that PD-1 expression is positively regulated by FTO and m^6^A mRNA methylation. m^6^A modification of the melanoma cell-intrinsic PD-1 (PDCD1) gene leads to increased mRNA decay via YTHDF2. PD-1 (PDCD1) expression is induced via the autophagy/NF-κB/FTO axis by metabolic stress and starvation, suggesting the FTO/PD-1 axis as a critical component of metabolic stress adaptation. Furthermore, FTO knockdown sensitized melanoma to anti-PD-1 blockade (Fig. 8a). Therefore, inhibiting the FTO pathway in melanoma may provide a new opportunity to reduce resistance to anti-PD-1 immunotherapy.

Functional intrinsic IFNγ signaling machinery is not only critical for promoting anti-tumor immunity^49^, but also required for the therapeutic response to immune checkpoint blockade^11^. Consistently we found that IFNγ downregulates FTO in melanoma cells, and FTO mediates resistance to melanoma cell killing by IFNγ through its m^6^A demethylase activity and downstream targets PD-1 (PDCD-1), CXCR4, and SOX10. FTO overexpression inhibits, while FTO knockdown promotes, IFNγ-induced cell death. It is possible that, at least in part, IFNγ exerts its cytotoxic effect by inhibiting FTO in melanoma cells. Indeed, anti-IFNγ antibody increased melanoma tumor growth in both control and FTO-knockdown cells in mice. Although FTO knockdown inhibits melanoma tumor growth in both immunocompromised and immunocompetent hosts (Figs. 2f, 8a, b), the response of FTO knockdown cells to anti-PD-1 requires host adaptive immunity (Fig. 8a, b). It appears that the melanoma intrinsic FTO pathway is not only critical for melanoma malignant traits and tumorigenicity in immunocompromised hosts, but also crucial for promoting resistance to IFNγ-induced killing in vitro and anti-PD-1 blockade in immunocompetent hosts in vivo. Although FTO knockdown in B16F10 increased the number of CD4+ TILs, it did not affect the number of IFNγ-producing CD4+ or CD8+ TILs (Fig. 8c, d). It is possible that the intrinsic function of the FTO pathway in IFNγ resistance mediated, at least in part, the effect of FTO knockdown in PD-1 blockade. Further studies are warranted to assess the full mechanism of immune regulation by PD-1 blockade with FTO inhibition in melanoma.

In summary, we have demonstrated that FTO inhibition suppresses melanoma tumorigenicity and the expression of melanoma cell-intrinsic genes PD-1 (PDCD1), CXCR4, and SOX10, at least partially through YTHDF2-mediated mRNA decay. We found that FTO inhibition enabled an anti-melanoma response to anti-PD-1 immunotherapy in mice. Our findings may provide new opportunities for developing improved melanoma therapies by inhibiting the FTO pathway in combination with anti-PD-1 immunotherapy.

## Methods

### Human skin tumor samples

All human specimens were studied after approval by the University of Chicago Institutional Review Board. The human melanoma array was obtained from US Biomax.

### Cell culture

WM35, WM115, WM793, and WM3670 were kindly provided by Dr. Meenhard Herlyn (Wistar Institute, Philadelphia, PA). Mel624, CHL-1, SK-mel30, and B16F10 cells were purchased from ATCC or provided by the Comprehensive Cancer Center Core Facilities at the University of Chicago. Melanoma cells were maintained in DMEM (Dulbecco’s modified Eagle’s medium) medium (Invitrogen) supplemented with fetal bovine serum (FBS, 10% HyClone,), penicillin (100 U/ml), and streptomycin (100 μg/ml, Invitrogen, Carlsbad, CA). NHEM cells (Normal Human Epidermal Melanocytes) were purchased form Lonza and cultured in 95% air and 5% CO_2_ at 37 °C in NHEM defined medium obtained from Lonza according to the manufacturer’s instructions.

### Lentiviral generation and infection

Lentiviral particles of shNC (Negative control) and shFTO for humans and mice were purchased from Santa Cruz Biotechnology. pLKO.1 plasmids of shNC, shFTO, shATG5, shATG7, and shYTHDF2 (human) were obtained from Sigma. pLenti plasmids of overexpression control (mGFP) and overexpression of PDCD1(mGFP-PDCD1), CXCR4, and SOX10 for humans were obtained from Origene (Origene, Rockville, MD). Lentivirus was produced by co-transfection into HEK-293T cells with lentiviral vectors in combination with the pCMVdelta8.2 packaging vectorand pVSV-G envelope vector using GenJet Plus DNA In Vitro Transfection Reagent (Signagen). Virus-containing supernatants were collected 24–48 h after transfection and used to infect recipients. Target cells were infected in the presence of Polybrene (8 μg/ml) (Sigma-Aldrich) and selected with puromycin (Santa Cruz Biotechnology, 1 μg/ml) for 6 days.

### Luciferase reporter assays

Using GenJet Plus DNA In Vitro Transfection Reagent (Signagen), cells were transfected with pGL3 NF-κB-Luc (1 μg) and pRL-TK (0.025 μg), as a control for transfection efficiency (Promega), according to the manufacturer’s instructions. Luciferase reporter assays (Promega) were carried out according to the manufacturer’s instructions.

### siRNA transfection

Using GenMute siRNA Transfection Reagent (Signagen), cells were transfected with siRNA (Dharmacon), targeting negative control (NC) METTL3, METTL14, YTHDF1, YTHDF2, and YTHDF3, according to the manufacturer’s instructions.

### Quantitative real-time PCR (qPCR)

qPCR assays were carried out using a CFX Connect real-time system (Bio-Rad, Hercules, CA) with Bio-Rad iQ SYBR Green Supermix (Bio-Rad). The threshold cycle number (CQ) was analyzed in triplicate for each sample. The CQ values for ANGPTL2, RAB40B, CTSV, RBBP9, NOP16, FCMR, PDCD1, CD274, CD47, CDKN1A, METTL3, METTL14, FTO, ALKBH5, YTHDF1, YTHDF2, YTHDF3, ATG5, ATG7, GAPDH, β-actin, and HPRT1 were normalized against HPRT1, GAPDH, or β-actin. The primer sequences used for qRT-PCR are shown in Supplementary Table 1.

### Flow cytometric analysis of apoptosis

Apopotosis was analyzed using the annexin V-FITC apoptosis detection kit (eBioscience), following the manufacturer’s instructions. Cell were then by a BD FACSCalibur flow cytometer (BD Biosciences). For cells viability assay in cells in suspension, poly-hydroxyethyl methacrylate (poly-HEMA) plates were used toculture cells in suspension. A solution of poly-HEMA (Sigma–Aldrich, St. Louis, MO) mixed in ethanol was poured onto polystyrene bacteriological dishes. After the poly-HEMA had dried, the same procedure was repeated once followed by extensive washing with phosphate buffered saline (PBS).

### Migration and invasion assay

For migration and invasion analysis, cell (5 × 10^4^) suspension (150 μl of serum-free medium) were seeded onto 8-mm Pore Transwell Inserts (Corning) coated with Matrigel for invasion assay, or without Matrigel for migration assay. Lower chambers were filled with complete medium (900 μl). Cells on the Transwell Inserts were then fixed with paraformaldehyde/PBS (4%) for 30 min. Next, fixed cells were stained with hematoxylin solution (Sigma-Aldrich) for 1 h. Then microphotograms of the cells migrated onto the lower side of the filter were imaged using a microscope. From the microphotograms, cells that migrated or invaded onto the lower side of the filter were manually counted. Cell numbers were quantified from ten randomly selected fields with the same area (500 μm × 500 μm) per Transwell insert.

### Soft agar colony formation and 3D on-top culture in matrigel

For soft agar assay, in a 35-mm petri dishes, 3000 cells were resuspended in 0.3% Agarose-low gelling temperature (Sigma-Aldrich) in DMEM with FBS (10%) and layered over 1 ml 0.5% agar in DMEM with 10% FBS. Colonies with more than eight cells were counted. For 3D on-top culture, 4-well glass chamber slides (BD Biosciences) were coated with 120 ml of growth factor reduced Matrigel per well; 5000 cells were seeded per well in DMEM with fetal bovine serum (10%) and Matrigel (10%).

### Immunofluorescence and immunohistochemistry

For immunofluorescence analysis, cells were first fixed with paraformaldehyde/PBS (4%) for 30 min, followed by permeabilized in Triton X-100/PBS (0.5%) for 20 min. Next cells were washed with PBS, blocked with PBS supplemented with 2% normal goat serum (Invitrogen) for 30 min, and stained for actin filaments with rhodamine-labeled phalloidin (Invitrogen, Carlsbad, CA). Then cells were washed three times with Triton X-100/TBS (0.1%) for 10 min and fixed in Prolong Gold Antifade with DAPI (Invitrogen), followed by observation under a fluorescence microscope (Olympus IX71).

For Human Melanoma Tissue Microarray staining, melanoma tissue array slides were obtained from US Biomax (Rockville, MD). After removing the blocking solution (3% albumin from chicken egg white (Sigma-Aldrich) in PBS), tissue array slides were incubated at 4 °C with primary rabbit anti-FTO, goat anti-PD-1, or mouse anti-MART1 for 18 h. After removing the primary antibodies, slides were washed with PBS solution with 0.025% TritonX-100. Tissue array slides were then incubated at room temperature with Alexa Fluor 594-conjugated secondary rabbit IgG (Jackson ImmunoResearch), Alexa Fluor 488-conjugated secondary mouse IgG (Jackson ImmunoResearch), and Alexa Fluor 405-conjugated secondary goat IgG (Jackson ImmunoResearch), then washed with TritonX-100 (0.025% in PBS), and mounted with Prolong Gold Antifade with DAPI (Invitrogen) forcell nucleus counterstaining for FTO/MART1 staining, or with Fluoromount Mounting Medium (Sigma-Aldrich) for FTO/PD-1/MART1 staining. Two investigators independently scored the immunofluorescence intensity and staining blindly, as 3 (strong), 2 (medium), 1 (weak), and 0 (negative). ImageJ (NIH) was also used for analyzing melanoma cells and tissue arrays. Areas with the same size were selected. The intensity of a selected area was measured followed by background subtraction to calculate mean pixel density. Stained samples were analyzed using a fluorescence microscope (Olympus IX71)

Antibodies used are as follows: anti-FTO (Abcam, ab126605, 1:100); anti-MART1 (NOVUS, NBP 2–15197, 1:100); Rhodamin-phalloidin (Life technologies, R415, 1:100); anti-PD-1 (R&D Systems, Cat# AF1086 Minneapolis, 1:100).

### Cell proliferation assay

Cell proliferation was assessed using the Cell Counting Kit-8 (CCK-8) (Sigma-Aldrich, St. Louis, MO) following the manufacturer’s protocol.

### Immunoblotting

Cell lysates were obtained using RIPA buffer (Pierce) containing inhibitors for proteases and phosphatases. Protein abundance were then analyzed by SDS-PAGE, and transferring onto nitrocellulose membranes followed by immunoblotting. Antibodies used are as follows:

Anti-ALKBH5 (Millipore Co., ABE 1013, 1:2000); anti-Beta-actin (Santa Cruz, SC-47778, 1:5000); anti-CXCR4 (Santa Cruz, SC-53534, 1:200); anti-CXCR4 (Novusbio, NBP1-77067SS, 1:5000); FTO (Santa Cruz, SC-271713, 1:200); GAPDH (Santa Cruz, SC-47724, 1:5000); GFP (Cell Signaling Technology, 2555S, 1:1000); LC3B (Cell Signaling Technology, 3868S, 1:1000); METTL14 (Millipore Co., ABE 1338, 1:1000); METTL3 (Proteintech, 15073-I-AP, 1:1000); p62 (Progen Biotechnik GmbH, GP62-C, 1:10,000); p70s6K (Cell Signaling Technology, 2708S, 1:2000); PD-1 (Proteintech, 66220-I-Ig, 1:5000); p-p70s6K (Cell Signaling Technology, 9234S, 1:1000); and SOX10 (Santa Cruz, SC-365692, 1:2000). The unprocessed blots are provided in Source Data.

### Mouse tumorigenesis and treatment

All of the animal procedures used were approved by the institutional animal care and use committee at the University of Chicago. Nude mice were purchased from Harlan Sprague-Dawley. C57BL/6 mice were obtained from Envigo. NSG (severely combined immunodeficient (NOD/SCID) interleukin-2 receptor (IL-2R) gamma chain null) mice were obtained from Jackson Laboratory. For xenograft experiments, one million cells were injected subcutaneously into the right flanks of 6-week-old female nude or C57BL/6 mice. Tumor growth was monitored and measured weekly by a caliper, and tumor volume was calculated using the formula, Tumor volume (mm^3^) = *d*^2^ × *D*/2, where *d* and *D* are the shortest and the longest diameters, respectively. For treatment with anti-PD-1 antibody (BioXCell, clone RMP1-14) or isotype control IgG antibody (BioXCell, clone 2A3), B16F10 melanoma cells (5 × 10^5^) were inoculated subcutaneously into C57BL/6 or NSG mice. When the tumors reached a volume of 80–100 mm^3^, mice were treated with anti-PD-1 or isotype control antibody (200 μg/mouse) by i.p. injection, every other day for three times. For IFNγ blockade treatment, C57BL/6 mice were treated with anti-IFNγ antibody (BioXcell, Clone XMG1.2) or isotype control IgG (BioXcell, Clone HRPN) (250 μg/mouse) every other day after tumor cell inoculation^50,51^.

### Analysis of tumor infiltrating lymphocytes (TILs)

Tumor tissue from B16F10 tumor-bearing mice (Day 14 after tumor cell inoculation) was dissociated by digestion with 2.5 mg/ml collagenase type IV (Worthington Biochemical, LS004188) and 100 μg/ml DNAse (Sigma-Aldrich, DN25) in RPMI 1640 with 5% FBS for 45 min at 37 °C. After digestion, tumor tissue was passed through 70-μm filters and mononuclear cells collected on the interface fraction between 40 and 80% per cell. Live cells (Zombie NIR negative) were gated using Zombie-violet (Catalog: 423105) staining. Next cells were gated using FSC-A and FSC-H to exclude doublets. Lymphocytes were gated on SSC-A and FSC-A. CD4^+^ and CD8^+^ TILs were gated on CD45^+^CD3^+^ cells. Gating strategies are shown in Supplementary Fig. 12a. The following mAbs recognizing the indicated antigens were used: FITC-anti-CD3 (Clone: 17A2, Catalog: 100204, 1:100), BV605-anti-CD4 (Clone: GK1.5, Catalog: 100451, 1:200), PE-Cy7-anti-CD8 (Clone: 53–6.7, Catalog: 100722, 1:200), PerCP-Cy5.5-anti-CD45 (Clone: 30-F11, Catalog: 103129, 1:400), Zombie-violet (Catalog: 423105), and APC-anti-IFNG (Clone: XMG1.2, Catalog: 505810, 1:100) (BioLegend). For assessment of IFNγ, cells were stimulated with 50 ng/ml phorbol 12-myristate 13-acetate (Sigma-Aldrich, P8139) and 1 μg/ml ionomycin (Fisher Scientific, BP25271) in the presence of Brefeldin A (BioLegend, 420601) for 4 h. After incubation, cells were then fixed. After surface staining, cell werepermeabilized using the BioLegend Kit (Catalog: 421002) and. Data were analyzed using FlowJo (version 10.5.3; FlowJo LLC).

### m^6^A dot blot assay

Total RNA was extracted using an RNeasy plus Mini Kit (QIAGEN, Hilden, Germany), following the manufacturer’s protocol. For mRNA isolation,first total RNA was extracted using an RNeasy mini kit with DNase I on-column digestion, followed by polyadenylated RNA extraction using a Dynabeads mRNA Purification Kit (Life technology, Carlsbad, CA). Then mRNA was concentrated with an RNA Clean & Concentrator-5 kit (Zymo Research, Irvine, CA). Briefly, RNA samples were loaded onto Amersham Hybond-N + membrane (GE Healthcare, Chicago, IL) and crosslinked to the membrane with UV radiation. Then the membrane was blocked with 5% nonfat dry milk (in 1X PBST) for 1–2 h, incubated with a specific anti-m^6^A antibody (Synaptic Systems, 202003, 1:2000) overnight at 4 °C followed by HRP-conjugated anti-rabbit IgG (Cell Signaling Technology) for 1 h at room temperature, and then developed with Thermo ECL SuperSignal Western Blotting Detection Reagent (Thermo Fisher Scientific, Waltham, MA).

### mRNA stability assay

A transcriptional inhibitor, actinomycin D (2 μM), inhibits mRNA transcription. Each sample was harvested at 0, 3, and 6 h after treatment with actinomycin D. Total RNA was isolated with an RNeasy plus mini kit (QIAGEN). The HPRT1 housekeeping gene was used as a loading control. HPRT1 mRNA does not contain m^6^A modifications, is not bound by YTHDF2, and is rarely affected by actinomycin D treatment^23,52^.

### m^6^A IP

100–150 μg total RNA was extracted from cells using TRIzol following the manufacturer’s protocol. mRNA was purified using a Dynabeads mRNA DIRECT Kit following the manufacturer’s protocols. One microgram mRNA was sonicated to ∼200 nt, 5% of fragmented mRNA was saved as input, and m^6^A containing mRNA fragments were enriched with an EpiMark N6-Methyladenosine Enrichment Kit following the manufacturer’s protocols. Finally, together with the input, IP RNA was extracted using RNA Clean and Concentrator (Zymo Research), followed by library preparation using the TruSeq stranded mRNA sample preparation kit (Illumina).

### Sequencing data analysis

General pre-processing of reads: sequencing were performed using Illumina Hiseq4000 with single end 80 bp read length. The adapters were removed by using cutadapt for m^6^A-seq, reads were aligned to the reference genome (hg38) in Tophat v2.0.14 using the parameter -g 1–library-type = fr-firststrand. RefSeq Gene structure annotations were obtained from the UCSC Table Browser. If a gene had multiple isoforms, the longest isoform was used. In order to eliminate the interference caused by introns in peak calling, aligned reads were extended to 150 bp (average fragment size) and converted from genome-based coordinates to isoform-based coordinates. The method used for peak calling was adopted from published work with modifications^17^. For calling m^6^A peaks, a gene’s longest isoform was scanned using a sliding window (100 bp) with a step of 10 bp. To mimimize bias from potential inaccuracy in gene structure annotation and/or the longest isoform, windows with read counts of less than 1/20 of the top window in both m^6^A-IP and the input were excluded. The read counts in each window for each gene were normalized by the median count of all windows forthe gene. The differential windows between IP and input samples were identified using a Fisher exact test. If the FDR < 0.01 and log2(Enrichment Score) ≥ 1, the window was considered as positive. Overlapping positive windows were merged. To obtain the enrichment score of each peak (or window), the following four numbers were calculated: (1) read counts of the IP samples in the current peak/window, (2) median read counts of the IP sample in all 100 bp windows on the current mRNA, (3) read counts of the input sample in the current peak/window, and (4) median read counts of the input sample in all 100 bp windows on the current mRNA. For each window, the enrichment score was calculated as (*a* × *d*)/(*b* × *c*).

### Gene-specific m^6^A qPCR

Real-time qPCR was performed to assess the relative abundance of the selected mRNA in m^6^A antibody IP samples and input samples. Briefly, total RNA was isolated with an RNeasy mini kit. With 500 ng RNA kept as an input sample, the remaining RNA was used for m^6^A-immunoprecipitation. Hundred microgram of RNA was diluted into 500 μl IP buffer (150 mM NaCl, 0.1% NP-40, 10 mM Tris, pH 7.4, 100 U RNase inhibitor) and incubated with m^6^A antibody (Synaptic Systems, Goettingen, Germany). The mixture was rotated at 4 °C for 2 h, then Dynabeads® Protein A (Thermo Fisher Scientific, Waltham, MA) coated with BSA was added into the solution and rotated for an additional 2 h at 4 °C. After washing by IP buffer with RNase inhibitors four times, the m^6^A IP portion was eluted with elution buffer (5 mM Tris-HCL pH 7.5, 1 mM EDTA pH 8.0, 0.05% SDS, and 4.2 μl Proteinase K (20 mg/ml)^18^. The final eluted mRNA was concentrated with an RNA Clean & Concentrator-5 kit (Zymo Research, Irvine, CA). The same amount of the concentrated IP RNA or input RNA from each sample was used for the cDNA library. The mRNA expression was determined by the number of amplification cycles (Cq). The relative m^6^A levels in genes were calculated by the m^6^A levels (m^6^A IP) normalized using the expression of each gene (Input).

### Microarray analysis

RNA was extracted from Mel624 cells with or without FTO knockdown with the RNeasy Mini Kit (Qiagen, Hilden, Germany), according to the manufacturer’s instructions. Total RNA concentration and purity were determined with NanoDrop (Thermo Scientific, Waltham, Mass), and total RNA integrity was confirmed with the Agilent Bioanalyzer (Agilent Technologies, Santa Clara, Calif). The RNA samples were processed with the HumanHT-12 v4.0 Gene Expression BeadChip microarray (Illumina) at the Functional Genomics Core Facility of the University of Chicago. The data have been deposited in the GEO repository with the accession numbers GSE128961.

### Statistical analyses

Statistical analyses were carried out using Prism 6 and 7 (GraphPad). Data were obtained from at least three independent experiments and for statistical significance were analyzed using Student’s *t*-test or Mann–Whitney *U*-test. *P* < 0.05 was considered statistically significant.

### Reporting summary

Further information on experimental design is available in the Nature Research Reporting Summary linked to this article.

## Supplementary information

Supplementary Information

Reporting Summary

Source Data

## Data Availability

m^6^A IP sequencing and RNA sequencing data are accessible at the GEO repository, under accession number GSE112902. Microarray data are accessible at the GEO repository, under accession number GSE128961. Other data from this study are available from the corresponding author upon request. The source data of immunoblots are provided as a Source Data file.

## References

[1] Tsao H, Chin L, Garraway LA, Fisher DE (2012). Melanoma: from mutations to medicine. Genes Dev..

[2] D’Orazio J, Jarrett S, Amaro-Ortiz A, Scott T (2013). UV radiation and the skin. Int. J. Mol. Sci..

[3] Leucci E (2016). Melanoma addiction to the long non-coding RNA SAMMSON. Nature.

[4] Luan W. et al. Long non-coding RNA MALAT1 acts as a competing endogenous RNA to promote malignant melanoma growth and metastasis by sponging miR-22. *Oncotarget* **7**, 63901 (2016). 10.18632/oncotarget.11564 PMC5325412 27564100

[5] Lian CG (2012). Loss of 5-hydroxymethylcytosine is an epigenetic hallmark of melanoma. Cell.

[6] Chapman PB (2011). Improved survival with vemurafenib in melanoma with BRAF V600E mutation. N. Engl. J. Med..

[7] Hodi FS (2010). Improved survival with ipilimumab in patients with metastatic melanoma. N. Engl. J. Med..

[8] Brahmer JR (2012). Safety and activity of anti-PD-L1 antibody in patients with advanced cancer. N. Engl. J. Med..

[9] Larkin J, Hodi FS, Wolchok JD (2015). Combined nivolumab and ipilimumab or monotherapy in untreated melanoma. N. Engl. J. Med..

[10] Spranger S, Gajewski TF (2018). Impact of oncogenic pathways on evasion of antitumour immune responses. Nat. Rev. Cancer.

[11] Sharma P, Hu-Lieskovan S, Wargo JA, Ribas A (2017). Primary, adaptive, and acquired resistance to cancer immunotherapy. Cell.

[12] Zhao BS, Roundtree IA, He C (2017). Post-transcriptional gene regulation by mRNA modifications. Nat. Rev. Mol. Cell Biol..

[13] Yue Y, Liu J, He C (2015). RNA N6-methyladenosine methylation in post-transcriptional gene expression regulation. Genes Dev..

[14] Fu Y, Dominissini D, Rechavi G, He C (2014). Gene expression regulation mediated through reversible m(6)A RNA methylation. Nat. Rev. Genet..

[15] Meyer KD, Jaffrey SR (2014). The dynamic epitranscriptome: N6-methyladenosine and gene expression control. Nat. Rev. Mol. Cell Biol..

[16] Jia G (2011). N6-methyladenosine in nuclear RNA is a major substrate of the obesity-associated FTO. Nat. Chem. Biol..

[17] Dominissini D (2012). Topology of the human and mouse m6A RNA methylomes revealed by m6A-seq. Nature.

[18] Meyer KD (2012). Comprehensive analysis of mRNA methylation reveals enrichment in 3’ UTRs and near stop codons. Cell.

[19] Batista PJ (2017). The RNA Modification N(6)-methyladenosine and Its Implications in Human Disease. Genom. Proteom. Bioinform..

[20] Wang S (2017). Roles of RNA methylation by means of N(6)-methyladenosine (m(6)A) in human cancers. Cancer Lett..

[21] Deng X (2018). RNA N(6)-methyladenosine modification in cancers: current status and perspectives. Cell Res..

[22] Robinson M, Shah P, Cui YH, He YY (2019). The role of dynamic m(6) A RNA methylation in photobiology. Photochem. Photobiol..

[23] Wang X (2014). N6-methyladenosine-dependent regulation of messenger RNA stability. Nature.

[24] Zhao X (2014). FTO-dependent demethylation of N6-methyladenosine regulates mRNA splicing and is required for adipogenesis. Cell Res..

[25] Wang X (2015). N(6)-methyladenosine modulates messenger RNA translation efficiency. Cell.

[26] Meyer KD (2015). 5’ UTR m(6)A promotes Cap-independent translation. Cell.

[27] Alarcon CR, Lee H, Goodarzi H, Halberg N, Tavazoie SF (2015). N6-methyladenosine marks primary microRNAs for processing. Nature.

[28] Liu N (2015). N (6)-methyladenosine-dependent RNA structural switches regulate RNA-protein interactions. Nature.

[29] Iles MM (2013). A variant in FTO shows association with melanoma risk not due to BMI. Nat. Genet..

[30] Li X (2013). Obesity-related genetic variants, human pigmentation, and risk of melanoma. Hum. Genet..

[31] Li Z (2017). FTO plays an oncogenic role in acute myeloid leukemia as a N6-methyladenosine RNA demethylase. Cancer Cell.

[32] Su R (2018). R-2HG exhibits anti-tumor activity by targeting FTO/m(6)A/MYC/CEBPA signaling. Cell.

[33] Cui Q (2017). m6A RNA methylation regulates the self-renewal and tumorigenesis of glioblastoma stem cells. Cell Rep..

[34] Fu Y (2013). FTO-mediated formation of N6-hydroxymethyladenosine and N6-formyladenosine in mammalian RNA. Nat. Commun..

[35] Kleffel S (2015). Melanoma cell-intrinsic PD-1 receptor functions promote tumor growth. Cell.

[36] Boussiotis VA (2016). Molecular and biochemical aspects of the PD-1 checkpoint pathway. N. Engl. J. Med..

[37] Ingram JR (2017). Localized CD47 blockade enhances immunotherapy for murine melanoma. Proc. Natl Acad. Sci. USA.

[38] Matlung HL, Szilagyi K, Barclay NA, van den Berg TK (2017). The CD47-SIRPalpha signaling axis as an innate immune checkpoint in cancer. Immunol. Rev..

[39] Vonderheide RH (2015). CD47 blockade as another immune checkpoint therapy for cancer. Nat. Med..

[40] Domanska UM (2013). A review on CXCR4/CXCL12 axis in oncology: no place to hide. Eur. J. Cancer.

[41] Tudrej KB, Czepielewska E, Kozlowska-Wojciechowska M (2017). SOX10-MITF pathway activity in melanoma cells. Arch. Med. Sci..

[42] Chatterjee S, Behnam Azad B, Nimmagadda S (2014). The intricate role of CXCR4 in cancer. Adv. Cancer Res..

[43] Chen S (2015). Combination of 4-1BB agonist and PD-1 antagonist promotes antitumor effector/memory CD8 T cells in a poorly immunogenic tumor model. Cancer Immunol. Res..

[44] Mauer J (2017). Reversible methylation of m(6)Am in the 5’ cap controls mRNA stability. Nature.

[45] Wei J (2018). Differential m(6)A, m(6)Am, and m(1)A demethylation mediated by FTO in the cell nucleus and cytoplasm. Mol. Cell.

[46] Akichika Shinichiro, Hirano Seiichi, Shichino Yuichi, Suzuki Takeo, Nishimasu Hiroshi, Ishitani Ryuichiro, Sugita Ai, Hirose Yutaka, Iwasaki Shintaro, Nureki Osamu, Suzuki Tsutomu (2018). Cap-specific terminal N6-methylation of RNA by an RNA polymerase II–associated methyltransferase. Science.

[47] Gulati P (2013). Role for the obesity-related FTO gene in the cellular sensing of amino acids. Proc. Natl Acad. Sci. USA.

[48] Postow MA, Callahan MK, Wolchok JD (2015). Immune checkpoint blockade in cancer therapy. J. Clin. Oncol..

[49] Dunn GP, Koebel CM, Schreiber RD (2006). Interferons, immunity and cancer immunoediting. Nat. Rev. Immunol..

[50] Yoshimura T (2008). Differential roles for IFN-gamma and IL-17 in experimental autoimmune uveoretinitis. Int. Immunol..

[51] Rynda-Apple A (2014). Regulation of IFN-gamma by IL-13 dictates susceptibility to secondary postinfluenza MRSA pneumonia. Eur. J. Immunol..

[52] Du H (2016). YTHDF2 destabilizes m(6)A-containing RNA through direct recruitment of the CCR4-NOT deadenylase complex. Nat. Commun..

