# Supplementary material for: m6A mRNA demethylase FTO regulates melanoma tumorigenicity and response to anti-PD-1 blockade
Source: Nat Commun. 2019 Jun 25;10:2782. doi: 10.1038/s41467-019-10669-0 (PMC6592937; doi:10.1038/s41467-019-10669-0)
Supplement: Supplementary file 1 — Supplementary Information [file 41467_2019_10669_MOESM1_ESM.pdf]

m^6A mRNA demethylase FTO regulates melanoma tumorigenicity and response to anti-PD-1 blockade

Yang et al.

Supplementary figures, figure legends, and table

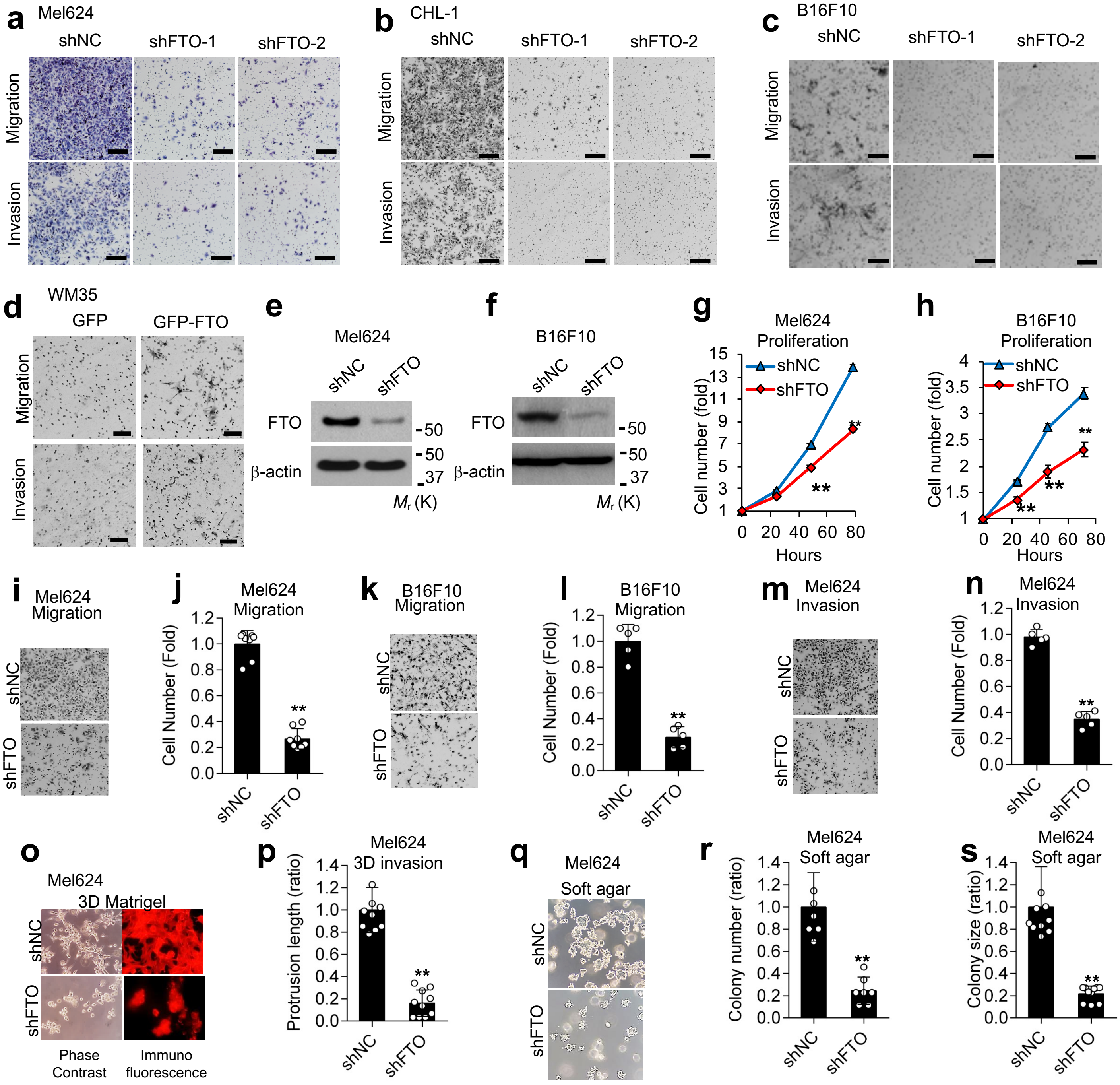

Supplementary Figure 1. Related to Figure 2. a-d Representative images of cell migration and invasion assay in Mel624 (**a**), CHL-1 (**b**), B16F10 (**c**), and WM35 (**d**) cells with or without FTO knockdown or forced overexpression. Scale bar: 100 μm. **e, f** Confirmation of FTO knockdown in Mel624 and B16F10 cells by immunoblot analysis, respectively. **g, h** Cell Proliferation analysis in Mel624 or B16F10 cells as in **e** and **f**, respectively. (**i-l**) Representative images and quantification of cell migration in Mel624 or B16F10 cells with or without FTO knockdown. **m, n** Representative images and quantification of cell invasion for Mel624 cells with or without FTO knockdown. **o** Representative images for phase contrast and immunofluorescence analysis for F-actin in Mel624 cells with or without FTO knockdown, cultured in 3D Matrigel. **p** Quantification of protrusion length for invasiveness under 3D Matrigel culture in Mel624 cells with or without FTO knockdown in **o**. **q** Representative images of colony-forming assay in Mel624 cells with or without FTO knockdown in soft agar. **r, s** Quantification of colony number (**r**) and size (**s**) in soft agar in cells in **q**. Data are shown as mean±S.D. (n≥3). **, P< 0.01; Student's *t*-test.

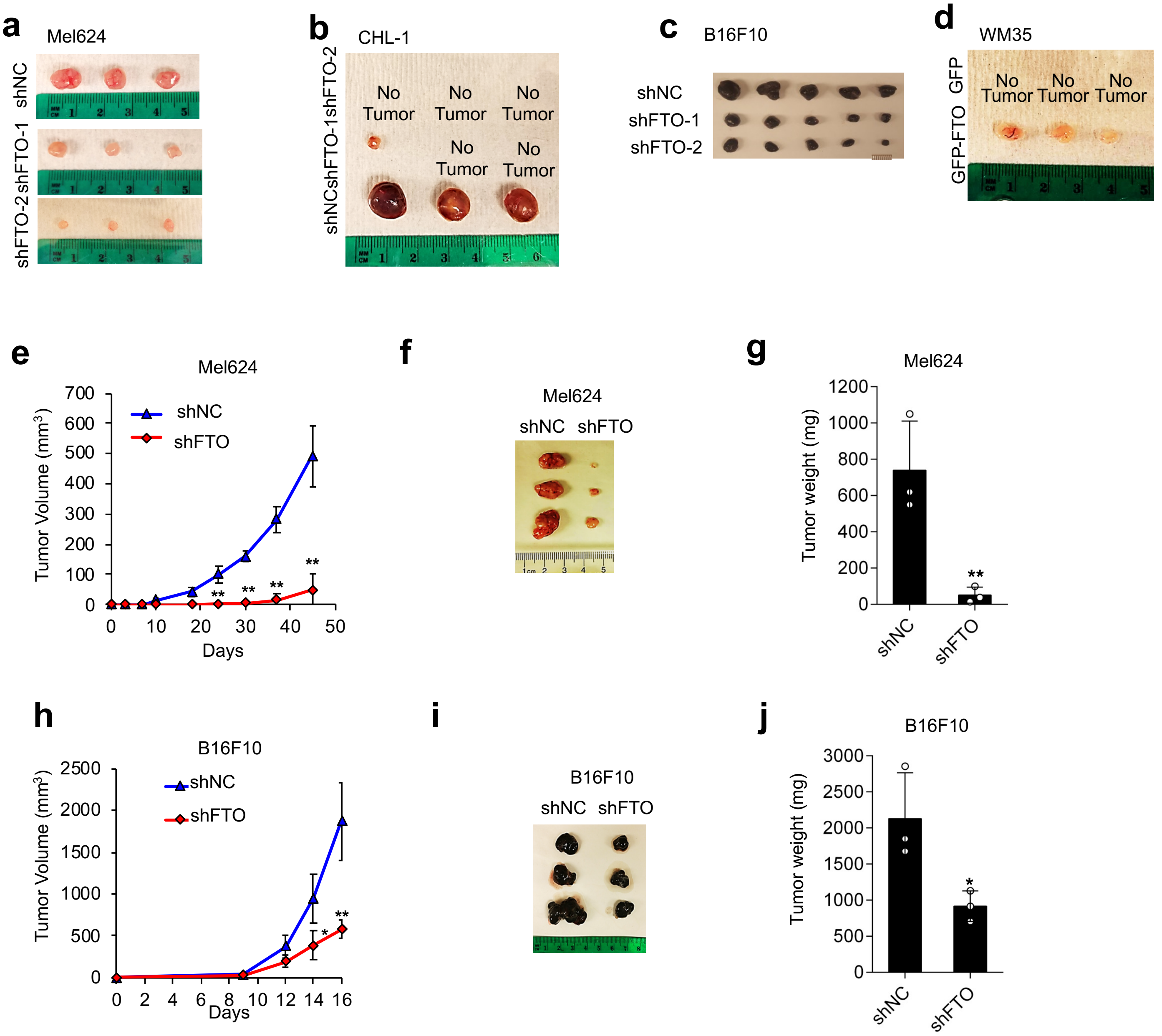

Supplementary Figure 2. Related to Figure 2.
a-d Representative images of tumors from Mel624 (**a**), CHL-1 (**b**), B16F10 (**c**), or WM35 (**d**) cells with or without FTO knockdown or forced overexpression after subcutaneous injection into nude mice (**a**, **b**, **d**) and C57BL/6 mice (**c**) (n=3). **e** Average tumor volume (mm^3) of Mel624 with or without FTO knockdown at different days after subcutaneous injection into nude mice (n = 3). **f** Image of tumors from Mel624 cells with or without FTO knockdown after subcutaneous injection into nude mice (n=3). **g** Final tumor weight from **f** (n = 3). **h** Average tumor volume (mm^3) of Mel624 with or without FTO knockdown at different days after subcutaneous injection into nude mice (n = 3). **i** Image of tumors from B16F10 cells with or without FTO knockdown after subcutaneous injection into C57BL/6 mice. **j** Final tumor weight from **i** (n = 3). Data are shown as mean±S.D. (n≥3). *, $P < 0.05$; **, P< 0.01; Student's *t*-test.

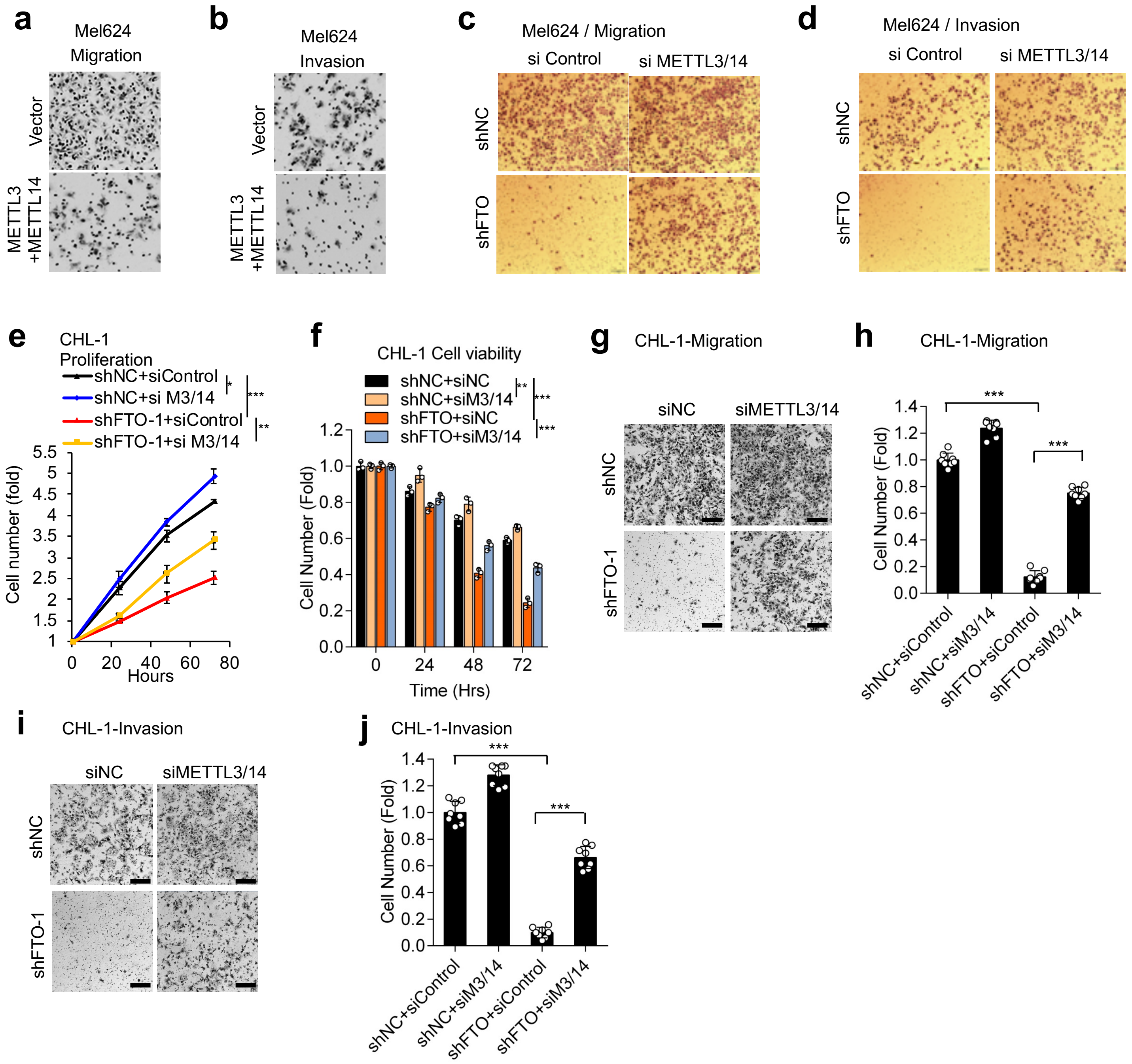

Supplementary Figure 3. Related to Figure 3.

a, b Representative images of migration and invasion assay in Mel624 cells transfected with control vector or the combination of METTL3-expressing and METTL14-expressing vectors. **c, d** Representative images of migration and invasion assay in Mel624 with or without FTO knockdown and/or the combination of METTL3 (M3) knockdown and METTL14 (M14) knockdown. **e-j** Proliferation (**e**), cell viability in cells in suspension (**f**), migration (**g, h**), and invasion analysis (**i, j**) in CHL-1 cells with or without FTO knockdown and/or the combination of M3 knockdown and M14 knockdown. Data are shown as mean±S.D. (n≥3). *, $P < 0.05$; **, $P < 0.01$; ***, $P < 0.001$; Student's *t*-test.

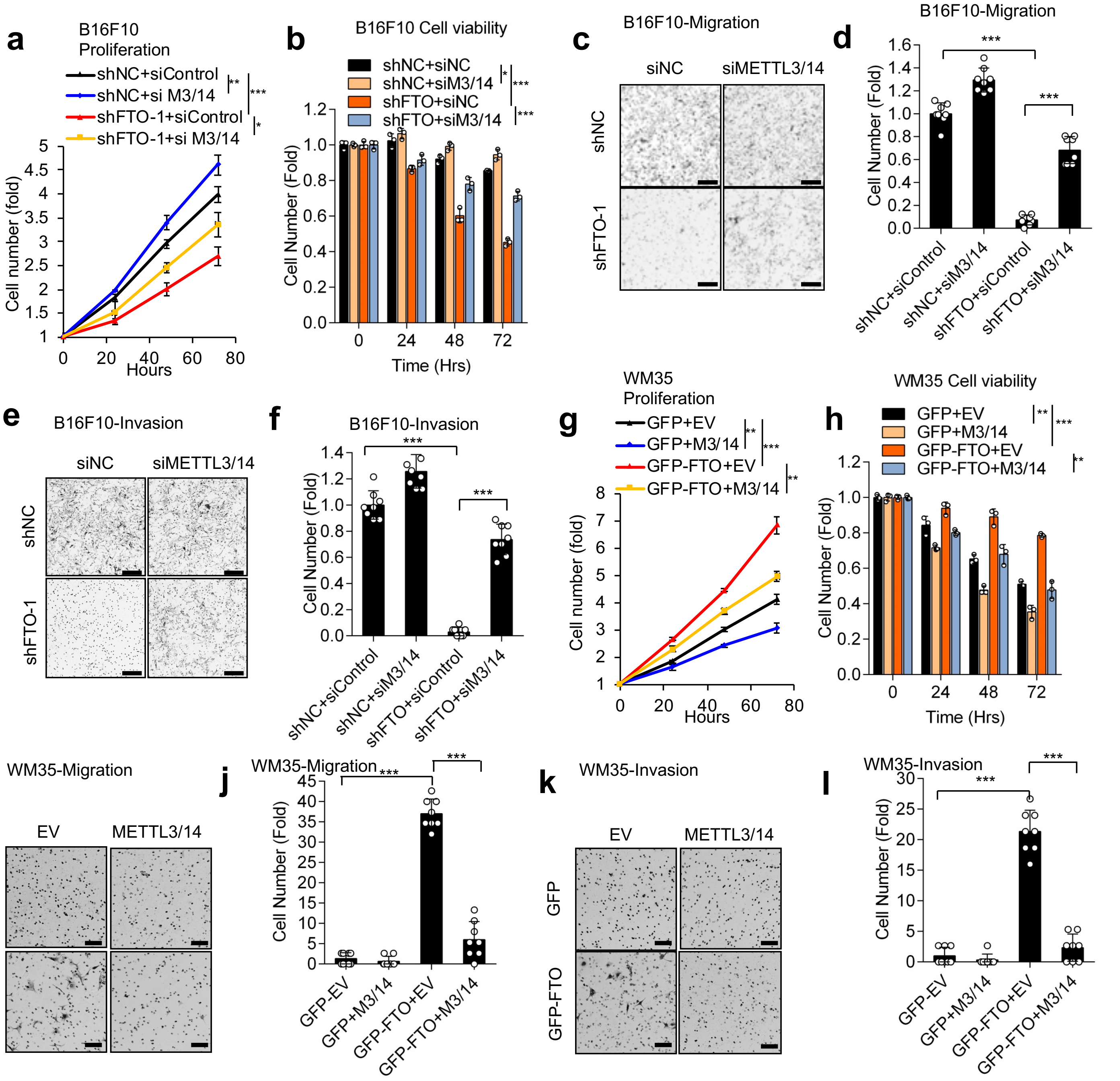

Supplementary Figure 4. Related to Figure 3.

a-f Proliferation (**a**), cell viability in cells in suspension (**b**), migration (**c, d**), and invasion (**e, f**) in B16F10 cells with or without FTO knockdown and/or the combination of M3 knockdown and M14 knockdown. **(g-l)** Proliferation assay (**g**), cell viability in cells in suspension (**h**), representative images (**i**) and analysis (**j**) of migration and representative images (**k**) and analysis (**l**) of invasion assay in WM35 cells with or without FTO overexpression and/or the combination of M3 overexpression and M14 overexpression. Scale bar: 100 μm. Data are shown as mean±S.D. (n≥3). *, P < 0.05; **, P< 0.01; ***, P < 0.001; Student's *t*-test.

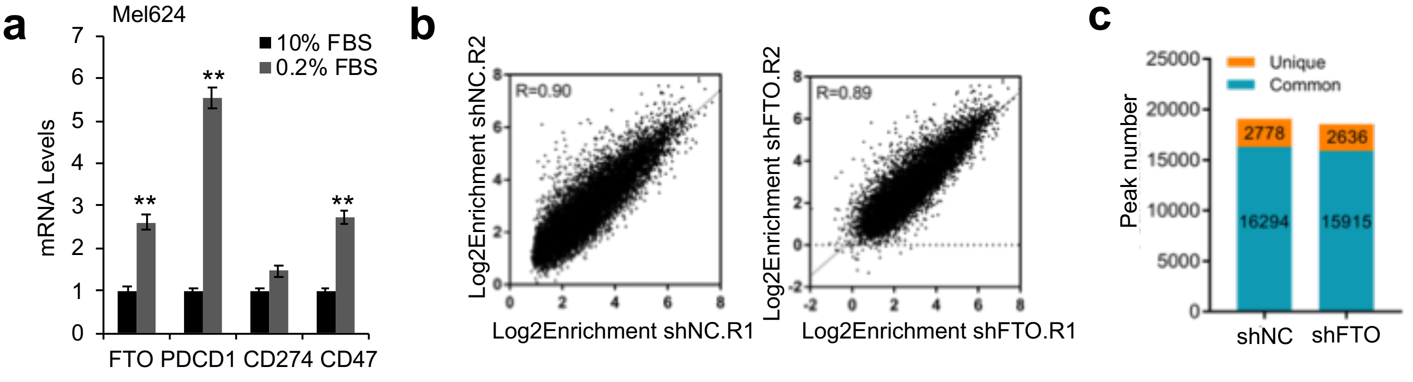

d

Relative mRNA levels (Ratio) in shFTO/shNC cells

	Microarray	RNA-seq
**PD-1 (PDCD1)**	ND	ND
**CXCR4**	0.6	0.2
**SOX10**	1.0	0.3

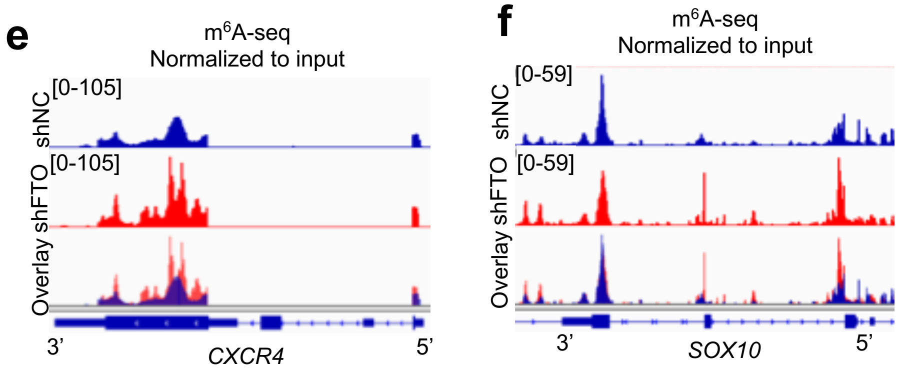

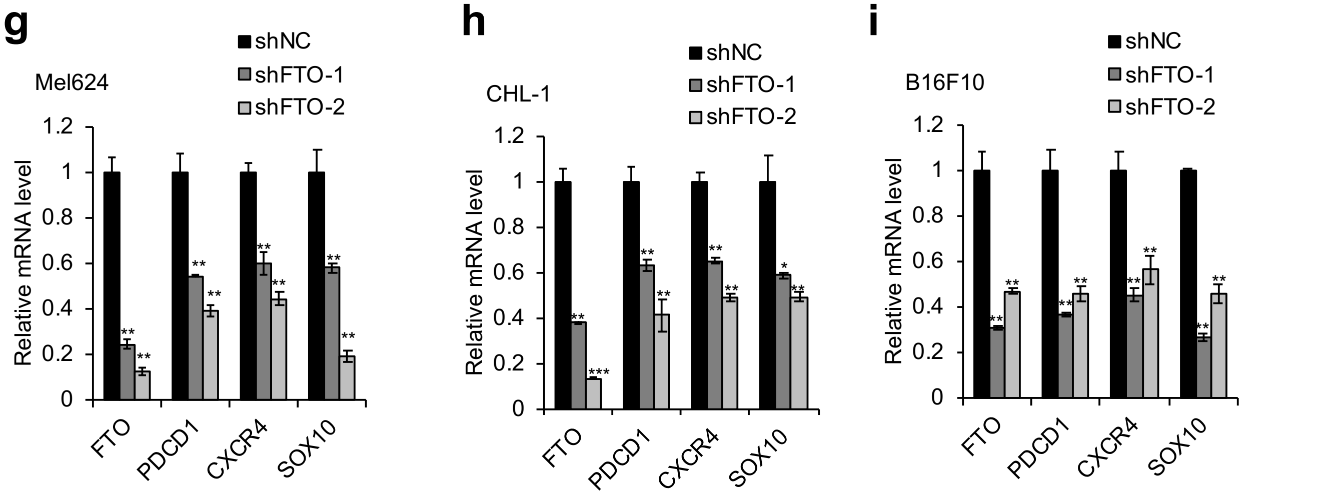

Supplementary Figure 5. Related to Figures 4 and 5.
a qPCR analysis of the mRNA levels of FTO, PD-1 (PDCD1), PD-L1 (CD274), and CD47 in Mel624 cells cultured with 10% FBS or 0.2% FBS. **b** Correlation of m^{6}A peaks between two independent samples from shNC (Left) and shFTO (Right) cells. **c** Number of m^{6}A peaks identified in m^{6}A-seq in shNC and shFTO Mel624 cells. Common m^{6}A genes contain at least one common m^{6}A peak, while unique m^{6}A genes contain no common m^{6}A peaks. **d** Ratio of gene expression in shFTO vs shNC Mel624 cells using microarray or RNA-seq analysis. ND, not detected. **e, f** Distribution of m^{6}A peaks across the CXCR4 **(e)** or SOX10 **(f)** mRNA transcripts. **g-i** qPCR analysis of mRNA levels in Mel624 (**g**), CHL-1 (**h**), and B16F10 (**i**) cells. Data are shown as mean±S.E. (n≥3). **, P< 0.01; ***, P < 0.001; Student's *t*-test.

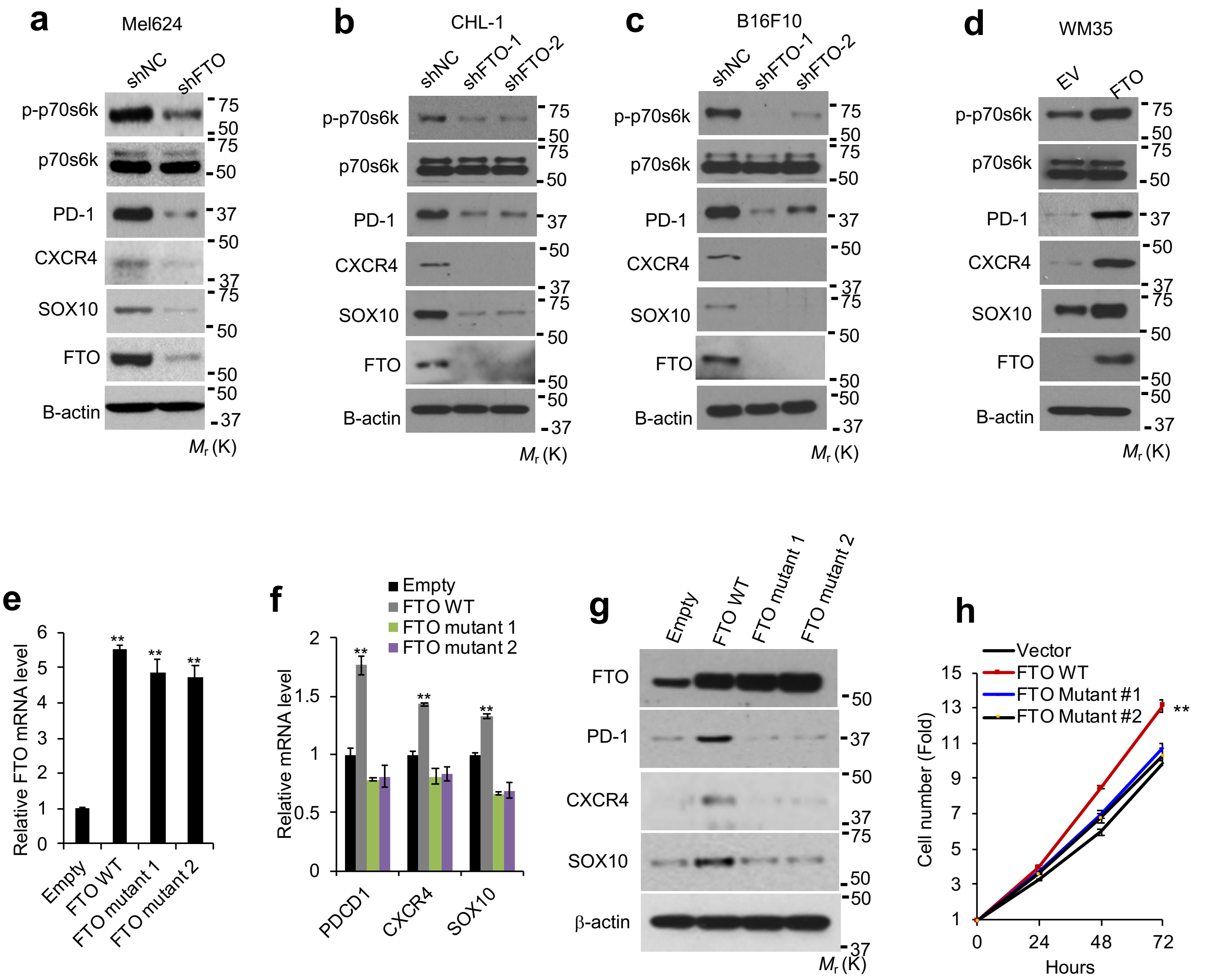

Supplementary Figure 6. Related to Figure 5.

a-c Immunoblot analysis of PD-1, p-p70S6K, p70S6K, CXCR4, SOX10, FTO and β-actin in Mel624, CHL-1, and B16F10 cells with or without knockdown of FTO. **d** Immunoblot analysis of PD-1, p-p70S6K, p70S6K, CXCR4, SOX10, FTO, and β-actin in WM35 cells with or without FTO overexpression. **e, f** qPCR analysis confirming overexpression of FTO WT, mutant 1 (H231A/D233A), and mutant 2 (R316Q/R322Q), and PDCD1, CXCR4, and SOX10 in Mel624 cells. **g** Immunoblot analysis of FTO, PD-1, CXCR4, SOX10, and β-actin in cells as in **f**. **h** Cell proliferation assay in cells as in **g**. Data are shown as mean±S.E. (**e, f**), and mean±S.D. (**h**) ($n \geq 3$). **, P< 0.01; Student's *t*-test.

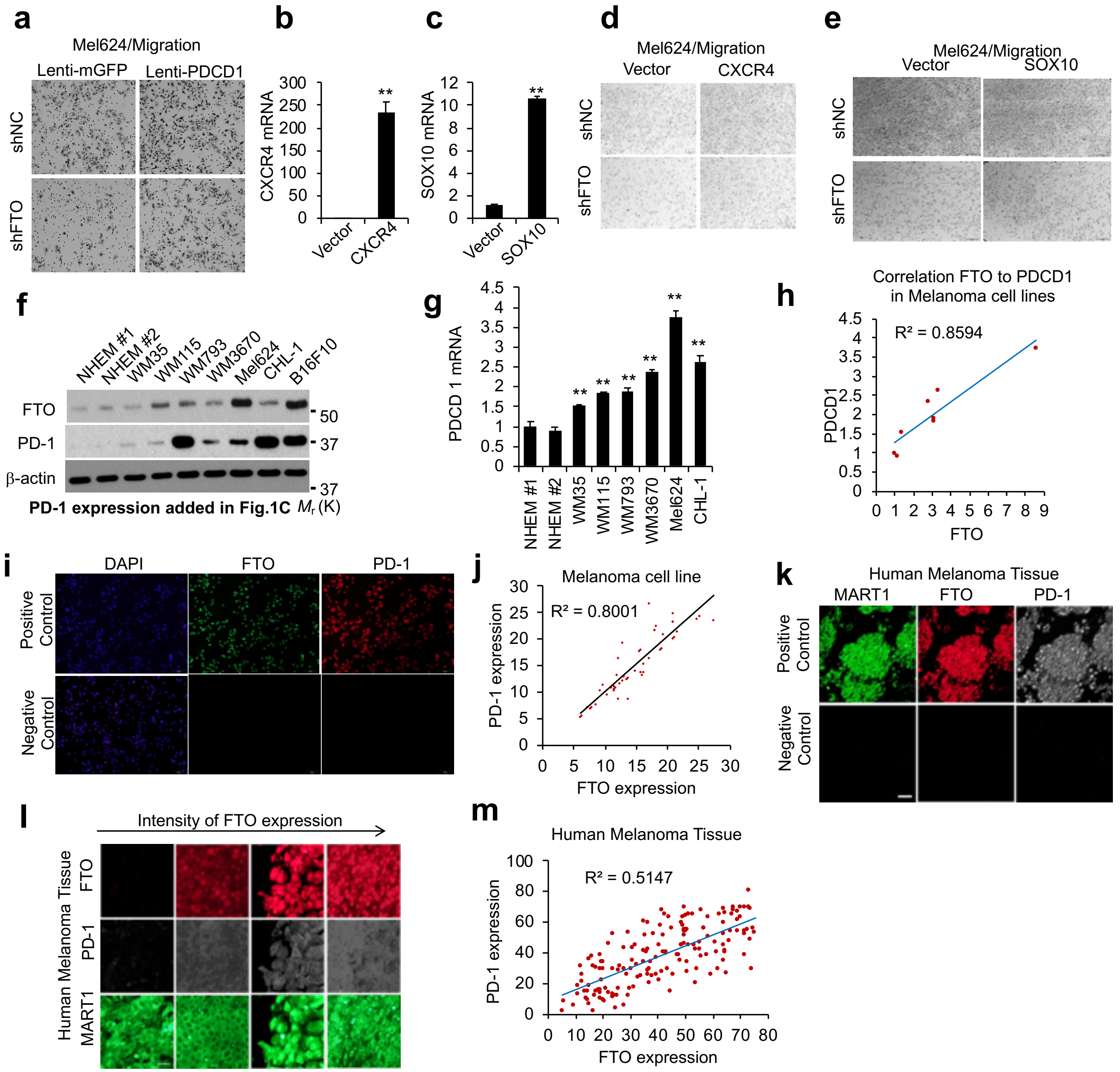

Supplementary Figure 7. Related to Figure 5.

a Representative images of cell migration assay of Mel624 cells with or without FTO knockdown, in combination with PD-1 (PDCD1) overexpression. **b, c** qPCR analysis of CXCR4 and SOX10 overexpression in Mel624 cells with or without forced overexpression of CXCR4 **(b)** and SOX10 **(c). d, e** Representative images of cell migration assay of Mel624 cells with or without FTO knockdown in combination with forced overexpression of CXCR4 (**d**) or SOX10 (**e**). **f** Additional immunoblot analysis of PD-1 to supplement the analysis in **Fig. 1c**. **g** qPCR analysis of the mRNA levels of PD-1/PDCD1 in normal melanocytes and melanoma cell lines. **h** Correlation analysis between FTO protein level and PD-1/PDCD1 mRNA levels. **i** Confirming the specificity of immunofluorescence analysis of FTO and PD-1s in melanoma cells. Negative control staining was performed without anti-FTO or anti-PD-1 primary antibodies. **j** Analysis using ImageJ for correlation between FTO and PD-1 protein level in melanoma cells. **k** Confirming the specificity of immunofluorescence analysis of FTO and PD-1 in human melanoma tissue as in **l**. Scale Bar: 20μm. **l** Representative images for the FTO and PD-1 protein levels in human melanoma samples. Scale Bar: 20μm. **m** Analysis using ImageJ for correlation between FTO and PD-1 protein levels in human melanoma tissue (n=164). Data are shown as mean±S.E. (n≥3). **, P< 0.01; Student's *t*-test.

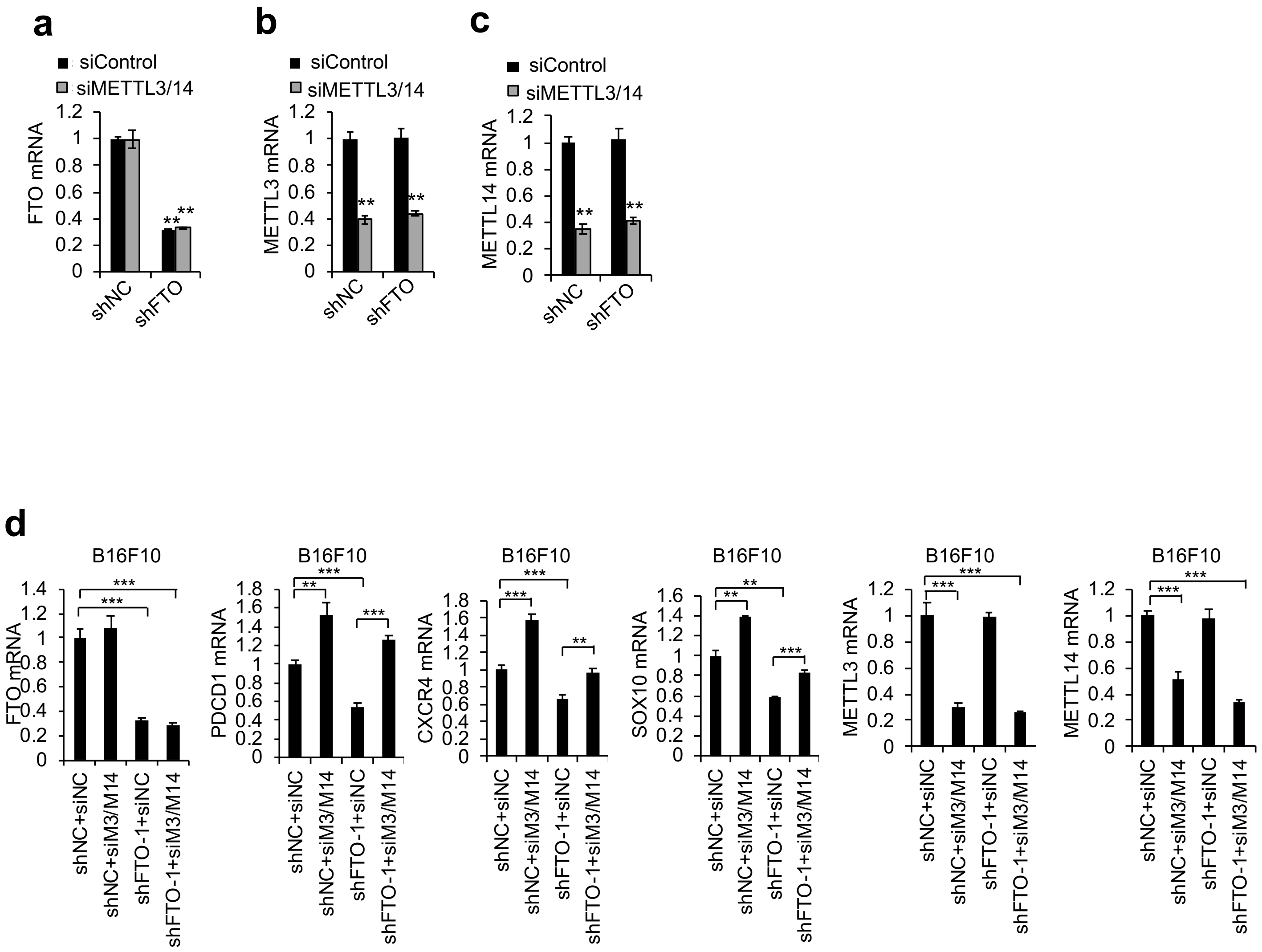

Supplementary Figure 8. Related to Figure 5.
a-c qPCR analysis confirming knockdown of FTO **(a)**, METTL3 **(b)**, and METTL14 **(c)** in Mel624 cells with or without FTO knockdown, in combination with siRNA knockdown of both METTL3 and METTL14. **d** qPCR analysis of mRNA for PDCD-1, CXCR4, and SOX10 in B16F10 with or without FTO. knockdown, in combination with siRNA knockdown of both METTL3 and METTL14. Data are shown as mean±S.E. (n≥3). **, P< 0.01; ***, P < 0.001; Student's *t*-test.

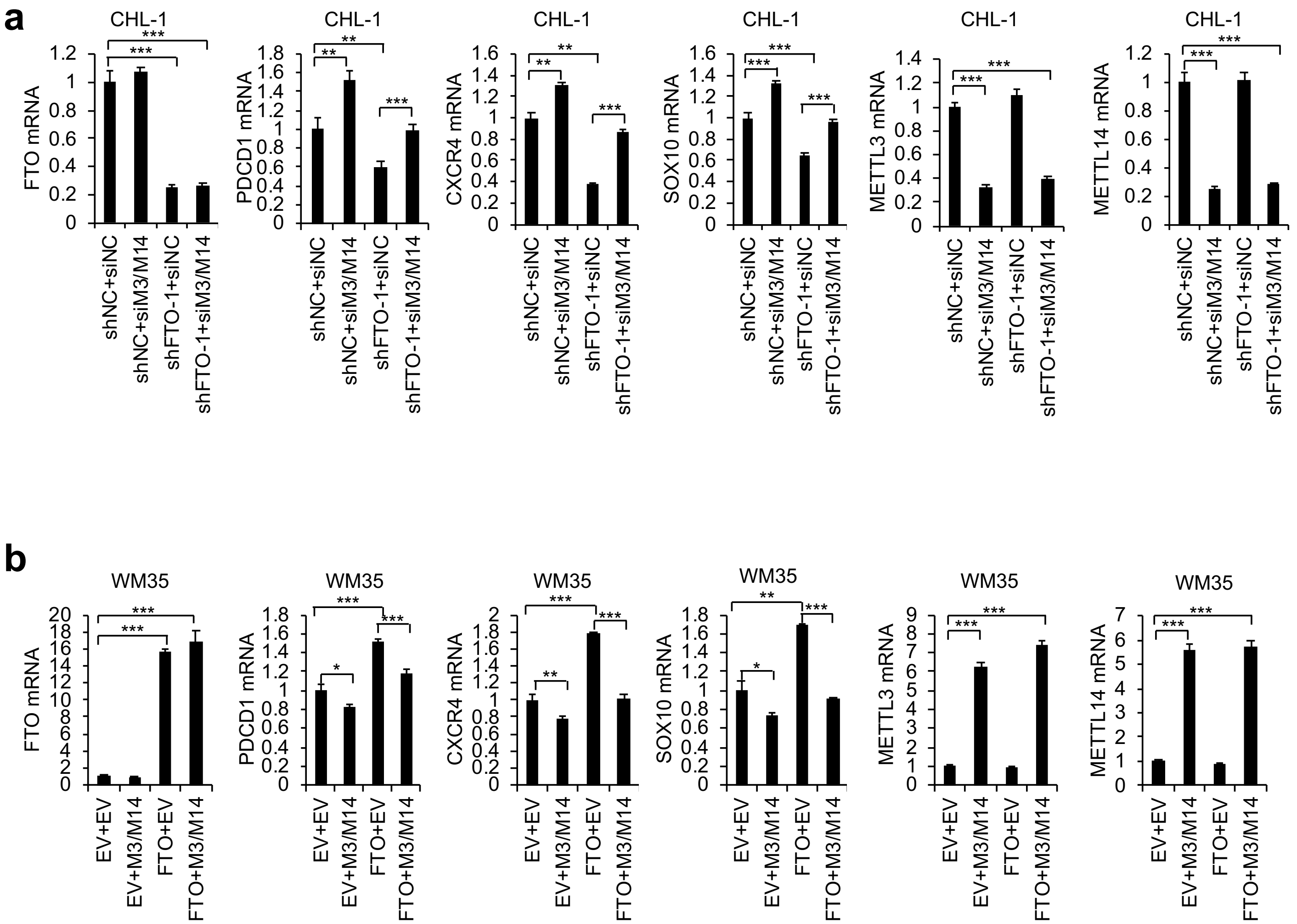

Supplementary Figure 9. Related to Figure 5.
a qPCR analysis of mRNA for PDCD-1, CXCR4, and SOX10 in CHL-1 cells with or without FTO knockdown, in combination with siRNA knockdown of both METTL3 and METTL14. **b** qPCR analysis of mRNA for PDCD-1, CXCR4, and SOX10 in WM35 cells with or without FTO overexpression, in combination with or without overexpression of both METTL3 and METTL14. Data are shown as mean±S.E. (n≥3). *, $P < 0.05$; **, $P< 0.01$; ***, $P < 0.001$; Student's *t*-test.

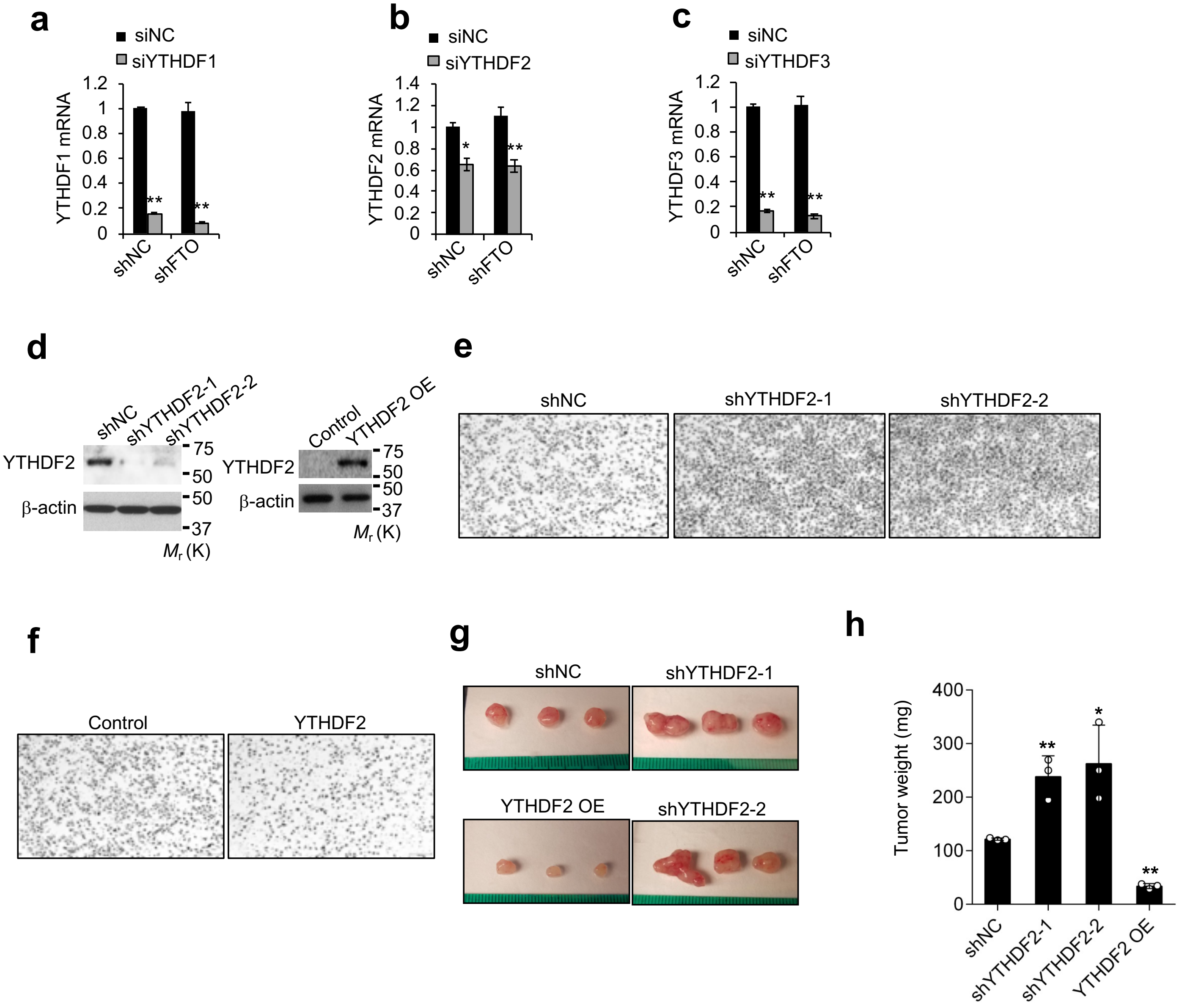

Supplementary Figure 10. Related to Figure 6.
a-c qPCR analysis of knockdown of YTHDF1-3 in Mel624 cells with or without FTO knockdown. **d** Immunoblot analysis confirming the knockdown or forced overexpression of YTHDF2 in Mel624 cells. **e, f** Representative images of cell migration analysis in Mel624 cells with or without YTHDF2 knockdown **(e)** or overexpression **(f)**. **g** Tumors from xenografted Mel624 cells with or without knockdown or forced overexpression of YTHDF2. **h** Final tumor weight from Mel624 cells with or without knockdown or forced overexpression of YTHDF2 (n = 3). Data are shown as mean±S.E. (**a-c**), and mean±S.D. (**h**) (n≥3). *, P < 0.05; **, P< 0.01; Student's *t*-test.

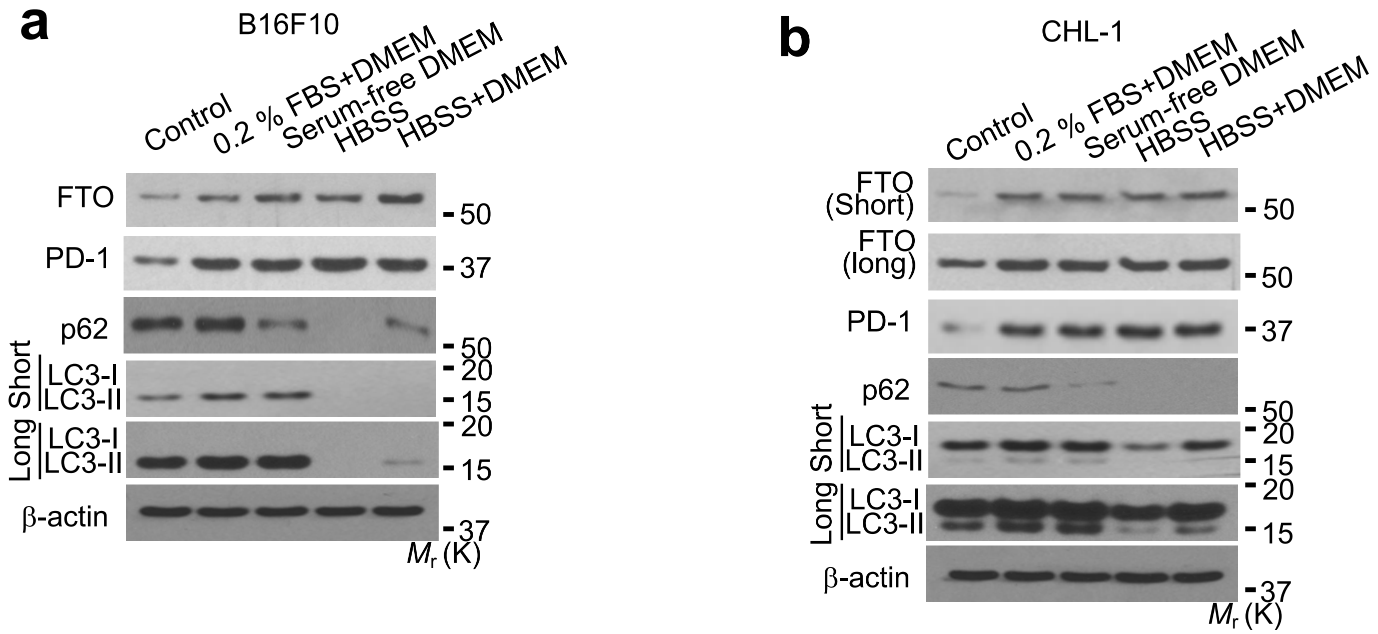

Supplementary Figure 11. Related to Figure 7.

a, b Immunoblot analysis of FTO, PD-1, p62, LC3-I/II (short and long exposure), and β-actin in B16F10 **(a)** and CHL-1 **(b)** cells cultured with control medium (10% FBS DMEM), 0.2% FBS DMEM, serum-free DMEM, Hanks' balanced salt solution containing calcium and magnesium (HBSS), or a combination of DMEM and HBSS (1:1 ratio).

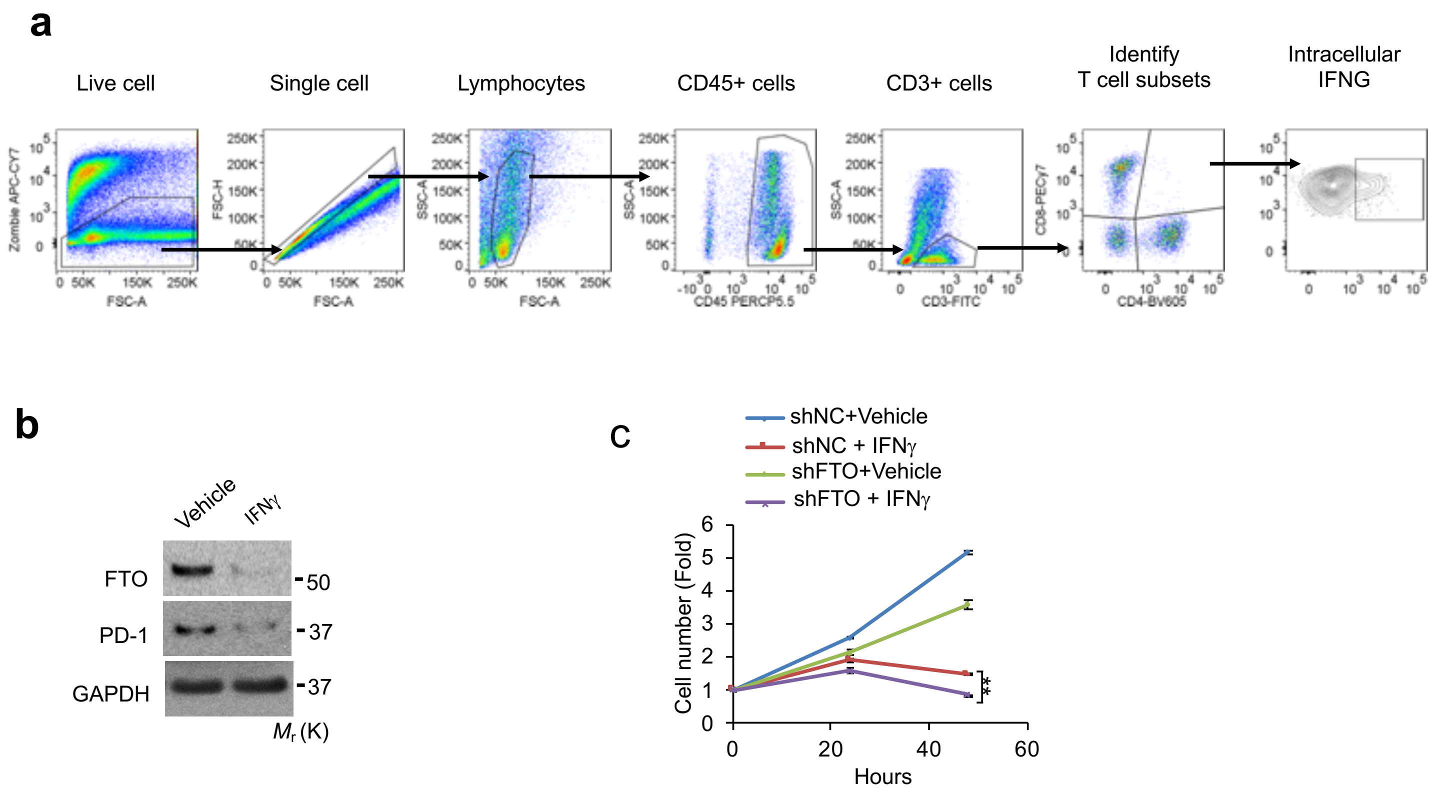

Supplementary Figure 12. Related to Figure 8.
a Gating strategy for Fig. 8C and 8D. **b** Immunoblot analysis of FTO, PD-1, and GAPDH in Mel624 cells treated with or without IFNγ (100 ng/ml) for 24 h. **c** Cell proliferation assay in Mel624 cells with or without FTO knockdown following treatment with or without IFNγ (50 ng/ml) for 48 h. Data are shown as mean±S.D. (n≥3). ** P< 0.01, Student's *t*-test.

Supplementary Table 1 Primers for qPCR analysis

Gene Name	Primer Direction	Primer Sequence
Human FCMR	Forward	5'- GGA TGG ACC TTG CAC TCT AG - 3'
	Reverse	5' – AGG CAT CTG GAA CAA ATA GGG - 3'
Human RAB40B	Forward	5' – GCA GCT CTG GGA TAC TTC AG - 3'
	Reverse	5' – GAT CAA TGC CGT CAA AAG ACC - 3'
Human CTSV	Forward	5' – CTG TTT CTT GAT CTT CCC AAA TCT G - 3'
	Reverse	5' – GAC AAG TTT CCC AGT TTT CCG - 3'
Human RBBP9	Forward	5' – CTA GCA AGG CAG TGA TTG TTC - 3'
	Reverse	5' – TCT CGT GCT GTA ATT GGG TC - 3'
Human NOP16	Forward	5' – ATC GAA TGC TCC CAC ATC C - 3'
	Reverse	5' – TCC CTA TGT CCA CCT CCA TG - 3'
Human ANGPTL2	Forward	5' – CCT GGA TGG CTC TGT TAA CTT C - 3'
	Reverse	5' – GTT TGT AGT TGC CTT GGT TCG - 3'
Human PDCD1 ref.5	Forward	5' – GAC AGC GGC ACC TAC CTC TGT G - 3'
	Reverse	5' – GAC CCA GAC TAG CAG CAC CAG G - 3'
Human PDCD1	Forward	5' – TGC TAG TCT GGG TCC TGG - 3'
	Reverse	5' – CAT AGT CCA CAG AGA ACA CAG G - 3'
Mouse PDCD1 ref.5	Forward	5' – CGG TTT CAA GGC ATG GTC ATT GG - 3'
	Reverse	5' – TCA GAG TGT CGT CCT TGC TTC C - 3'
Mouse PDCD1	Forward	5' – GGT ACC CTG GTC ATT CAC TTG - 3'
	Reverse	5' – ATT TGC TCC CTC TGA CAC TG - 3'
Human CD274	Forward	5' – TCA CTT GGT AAT TCT GGG AGC - 3'
	Reverse	5' – CTT TGA GTT TGT ATC TTG GAT GCC - 3'
Human CD47	Forward	5' – TTT TGC TAT ACT CCT GTT CTG GG - 3'
	Reverse	5' – TGG GAC GAA AAG AAT GGC TC - 3'
Human YTHDF3	Forward	5' – TGC ACA TTA TGA AAA GCG TCA AG - 3'
	Reverse	5' – GGC ATT TCC AGA GTC TAC ATC G - 3'
Human FTO	Forward	5' – ACT TGG CTC CCT TAT CTG ACC - 3'
	Reverse	5' – TGT GCA GTG TGA GAA AGG CTT - 3'
Mouse FTO	Forward	5' – TCA CAG CCT CGG TTT AGT TC- 3'
	Reverse	5' – GCA GGA TCA AAG GAT TTC AAC G - 3'
Human YTHDF2	Forward	5' – TCT GGA AAA GGC TAA GCA GG - 3'
	Reverse	5' – CTT TTA TTT CCC ACG ACC TTG AC - 3'
Human YTHDF1	Forward	5' – CAC AAC CTC CAT CTT CGA C - 3'
	Reverse	5' – ACA CAA CCT CCA TCT TCG AC - 3'
Human ALKBH5	Forward	5' – CCC TGC TCT GAA ACC CAA G - 3'
	Reverse	5' – GTT CTC TTC CTT GTC CAT CTC C - 3'
Human METTL14	Forward	5' – TTT CTC TGG TGT GGT TCT GG - 3'
	Reverse	5' – AAG TCT TAG TCT TCC CAG GAT TG - 3'
Human METTL3	Forward	5' – GAA AGA CTA TCT CCT GGC ACT C - 3'
	Reverse	5' – GTA CCT TTG CTT GAA CCG TG - 3'
Human GAPDH	Forward	5' – AAT CCC ATC ACC ATC TTC CA - 3'
	Reverse	5' – TGG ACT CCA CGA CGT ACT CA - 3'
Human b-actin	Forward	5' – ACC TTC TAC AAT GAG CTG CG - 3'
	Reverse	5' – CCT GGA TAG CAA CGT ACA TGG - 3'
Mouse b-actin	Forward	5' – ACC TTC TAC AAT GAG CTG CG- 3'
	Reverse	5' – CTG GAT GGC TAC GTA CAT GG - 3'
Human HPRT1	Forward	5' – TGC TGA GGA TTT GGA AAG GG - 3'
	Reverse	5' – ACA GAG GGC TAC AAT GTG ATG - 3'
