# Supplementary material for: m6A mRNA demethylase FTO regulates melanoma tumorigenicity and response to anti-PD-1 blockade
Source: Nat Commun. 2019 Jun 25;10:2782. doi: 10.1038/s41467-019-10669-0 (PMC6592937; doi:10.1038/s41467-019-10669-0)
Supplement: Supplementary file 2 — Reporting Summary [file 41467_2019_10669_MOESM2_ESM.pdf]

nature research

Corresponding author(s): Yu-Ying He, Chuan He

Last updated by author(s): 2019/05/06

Reporting Summary

Nature Research wishes to improve the reproducibility of the work that we publish. This form provides structure for consistency and transparency in reporting. For further information on Nature Research policies, see Authors & Referees and the Editorial Policy Checklist.

Statistics

For all statistical analyses, confirm that the following items are present in the figure legend, table legend, main text, or Methods section.

n/a	Confirmed	
☐	☒	The exact sample size (*n*) for each experimental group/condition, given as a discrete number and unit of measurement
☐	☒	A statement on whether measurements were taken from distinct samples or whether the same sample was measured repeatedly
☐	☒	The statistical test(s) used AND whether they are one- or two-sided<br>*Only common tests should be described solely by name; describe more complex techniques in the Methods section.*
☒	☐	A description of all covariates tested
☐	☒	A description of any assumptions or corrections, such as tests of normality and adjustment for multiple comparisons
☐	☒	A full description of the statistical parameters including central tendency (e.g. means) or other basic estimates (e.g. regression coefficient) AND variation (e.g. standard deviation) or associated estimates of uncertainty (e.g. confidence intervals)
☐	☒	For null hypothesis testing, the test statistic (e.g. *F*, *t*, *r*) with confidence intervals, effect sizes, degrees of freedom and *P* value noted<br>*Give P values as exact values whenever suitable.*
☒	☐	For Bayesian analysis, information on the choice of priors and Markov chain Monte Carlo settings
☒	☐	For hierarchical and complex designs, identification of the appropriate level for tests and full reporting of outcomes
☒	☐	Estimates of effect sizes (e.g. Cohen's *d*, Pearson's *r*), indicating how they were calculated

Our web collection on statistics for biologists contains articles on many of the points above.

Software and code

Policy information about availability of computer code

Data collection	For Immunofluorescence analaysis: Cell Sens standard Ver.1.17<br>For mRNA level analysis : Bio-Rad CFX Manager
Data analysis	For Statistical analysis: GraphPad Prism 5 (Ver. 6.07), Prism 7 (Ver. 7.0d), and Microsoft excel 2010<br>For Flow Cytometry analysis : BD CellQuest (TM)- Ver. 5.2 and FlowJo software (version 10.5.3; FlowJo LLC)<br>For Soft agar colony formation and 3D on-top culture in Matrigel: ImageJ ver 1.46r<br>Sequencing analysis: the adapters were removed by using cutadapt for m6A-seq, reads were aligned to the reference genome (hg38) using Tophat v2.0.14 with parameter -g 1 --library-type=fr-firststrand. RefSeq Gene structure annotations were downloaded from UCSC Table Browser.

For manuscripts utilizing custom algorithms or software that are central to the research but not yet described in published literature, software must be made available to editors/reviewers. We strongly encourage code deposition in a community repository (e.g. GitHub). See the Nature Research guidelines for submitting code & software for further information.

Data

Policy information about availability of data

All manuscripts must include a data availability statement. This statement should provide the following information, where applicable:

- Accession codes, unique identifiers, or web links for publicly available datasets
- A list of figures that have associated raw data
- A description of any restrictions on data availability

The data have been deposited in the GEO repository with the accession numbers GSE112902 (https://www.ncbi.nlm.nih.gov/geo/query/acc.cgi?acc=GSE112902). Microarray data are accessible at the GEO repository, under accession number GSE128961 (https://www.ncbi.nlm.nih.gov/geo/query/acc.cgi?acc=GSE128961).

Field-specific reporting

Please select the one below that is the best fit for your research. If you are not sure, read the appropriate sections before making your selection.

☒ Life sciences ☐ Behavioural & social sciences ☐ Ecological, evolutionary & environmental sciences

For a reference copy of the document with all sections, see nature.com/documents/nr-reporting-summary-flat.pdf

Life sciences study design

All studies must disclose on these points even when the disclosure is negative.

Sample size	Generally no statistical analysis was performed to predetermine the sample size. Sample size and number of animals are selected based on our previous experiences of carrying out similar experiments and published work. Sample size and number of independent experiments are clearly stated in the figure legend or in the Methods section. Three to more independent replicates were used to perform statistical analyses.
Data exclusions	No data were excluded from analysis.
Replication	Experiments were repeated two to three times independently. Replication were described in the figure legends.
Randomization	For the xenograft model, animals were randomly assigned into groups receiving various cell line injections. Randomization (formal or otherwise) was not relevant for other data included in the manuscript.
Blinding	For immunofluorescence (IF) experiment, blind staining and blind analysis were carried out. For other experiments, the investigators were not blinded to group allocation during data collection and/or analysis.

Reporting for specific materials, systems and methods

We require information from authors about some types of materials, experimental systems and methods used in many studies. Here, indicate whether each material, system or method listed is relevant to your study. If you are not sure if a list item applies to your research, read the appropriate section before selecting a response.

Materials & experimental systems

n/a	Involved in the study	
☐	☒	Antibodies
☐	☒	Eukaryotic cell lines
☒	☐	Palaeontology
☐	☒	Animals and other organisms
☒	☐	Human research participants
☒	☐	Clinical data

Methods

n/a	Involved in the study	
☒	☐	ChIP-seq
☐	☒	Flow cytometry
☒	☐	MRI-based neuroimaging

Antibodies

Antibodies used

ALKBH5 Millipore Co. Cat.# ABE 1013 Bedford, MA (1:2000 for WB)
Beta-actin Santa Cruz Cat.# SC-47778 Santa Cruz, CA (1:5000 for WB)
CXCR4 Santa Cruz Cat.# SC-53534 Santa Cruz, CA (1:200 for WB)
CXCR4 Novusbio # NBP1-77067SS (1:5000 for WB)
FTO Santa Cruz Cat. # SC-271713 Santa Cruz, CA (1:200 for WB)
FTO (Immunofluorescence) Abcam Cat. # ab126605 Cambridge, United Kingdom (1:100 for IF)
GAPDH Santa Cruz Cat. # SC-47724 Santa Cruz, CA (1:5000 for WB)
GFP Cell Signaling Technology Cat. # 2555S Beverly, MA (1:1000 for WB)
LC3B Cell Signaling Technology Cat. # 3868S Beverly, MA (1:1000 for WB)
m6A Synaptic system Cat. # 202 003 Goettingen, Germany (1:2000 for WB and 1:200 for IP)
MART1 (Immunofluorescence) NOVUS Cat. # NBP 2-15197 St. Louis, MO (1:100 for IF)
METTL14 Millipore Co. Cat. # ABE 1338 Bedford, MA (1:1000 for WB)
METTL3 Proteintech Cat. # 15073-I-AP Chicago, IL (1:1000 for WB)
p62 Progen Biotechnik GmbH Cat. # GP62-C Heidelberg, Germany (1:10000 for WB)
p70s6K Cell Signaling Technology Cat. # 2708S Beverly, MA (1:2000 for WB)
PD-1 Proteintech Cat. # 66220-I-Ig Chicago, IL (1:5000 for WB)
p-p70s6K Cell Signaling Technology Cat. # 9234S Beverly, MA (1:1000 for WB)
Rhodamin-phalloidin Life technologies Cat. # R415 Carlsbad, CA (1:100 for IF)
SOX10 Santa Cruz Cat. # SC-365692 Santa Cruz, CA (1:2000 for WB)
PD-1 (Immunofluorescence) R & D Systems Cat# AF1086 Minneapolis, MN (1: 100 for IF)
FITC-anti-CD3 Clone:17A2 BioLegend Cat# 100204, San Diego, CA (1:100 for Flow Cyt)
BV605-anti-CD4 Clone: GK1.5, BioLegend Cat# 100451, San Diego, CA (1:200 for Flow Cyt)

PE-Cy7-anti-CD8 Clone:53-6.7, BioLegend Cat#100722, San Diego, CA (1:200 for Flow Cyt)
PerCP-Cy5.5-anti-CD45 Clone:30-F11, BioLegend Cat#103129, San Diego, CA (1:400 for Flow Cyt)
APC-anti-IFNG Clone:XMG1.2, BioLegend Cat#505810, San Diego, CA (1:100 for Flow Cyt)
Blocking antibody: anti-mouse PD-1 (CD279) BioXcell; InVivoMAb, Cat# BE0146, West Lebanon, NH (200ug/mouse)
Blocking antibody: isotype control, anti-trinitrophenol BioXcell; InVivoMAb, Cat# BE0089, West Lebanon, NH (200ug/mouse)
Blocking antibody: InVivoMAb anti-mouse IFNγ, Cat# BE0055, West Lebanon, NH (250ug/mouse)
Blocking antibody: InVivoMAb rat IgG1 isotype control, anti-horseradish peroxidase Cat# BE0088, West Lebanon, NH (250ug/mouse)
Alexa Fluor 594-conjugated secondary rabbit IgG, Jackson ImmunoResearch, Cat# 111-585-003, West Grove, PA (1: 100 for IF)
Alexa Fluor 594-conjugated secondary rabbit IgG, Jackson ImmunoResearch, Cat# 711-585-152, West Grove, PA (1: 100 for IF)
Alexa Fluor 488-conjugated secondary mouse IgG, Jackson ImmunoResearch, Cat# 115-545-166, West Grove, PA (1: 100 for IF)
Alexa Fluor 488-conjugated secondary mouse IgG, Jackson ImmunoResearch, Cat# 715-545-150, West Grove, PA (1: 100 for IF)
Alexa Fluor 405-conjugated secondary goat IgG, Jackson ImmunoResearch, Cat# 705-475-003, West Grove, PA (1: 100 for IF)

Validation

The commercial antibodies were validated based on the information of the manufacturers' instructions and additionally the antibodies were validated by the use of negative control and/or positive control (such as knockdown or overexpression) for FTO, PD-1, METTL3, METTL14 and GFP antibodies.
The concentration recommended from antibody's date sheet was used for western blot, dot blot and immunostaining.

Eukaryotic cell lines

Policy information about cell lines

Cell line source(s)

WM35, WM115, WM793, WM3670 were kindly provided by Dr. Meenhard Herlyn (Wistar Institute, Philadelphia, PA). Mel624, CHL-1, SK-mel30, and B16F10 cells were obtained from ATCC and University of Chicago Comprehensive Cancer Center Core Facilities.

Authentication

All cell lines were authenticated according to the ATCC cell line authentication test recommendations, including morphology check by microscope, growth curve analysis, and mycoplasma test.

Mycoplasma contamination

All cell lines were tested to be mycoplasma negative. All lines were routinely tested for mycoplasma contamination.

Commonly misidentified lines
(See ICLAC register)

No commonly misidentified cell lines listed by ICLAC were used.

Animals and other organisms

Policy information about studies involving animals; ARRIVE guidelines recommended for reporting animal research

Laboratory animals

Athymic Nude mice (6-8 weeks of age) were obtained from Harlan Sprague-Dawley.
C57BL/6 mice (6-8 weeks of age) were obtained from Envigo.
NSG mice (severely combined immunodeficient (NOD/SCID) interleukin-2 receptor (IL-2R) gamma chain null) were obtained from Jackson Laboratory.

Wild animals

No wild animal was used in this research.

Field-collected samples

No field-collected samples were used.

Ethics oversight

All animal procedures used were approved by the University of Chicago institutional animal care and use committee

Note that full information on the approval of the study protocol must also be provided in the manuscript.

Flow Cytometry

Plots

Confirm that:

☒ The axis labels state the marker and fluorochrome used (e.g. CD4-FITC).

☒ The axis scales are clearly visible. Include numbers along axes only for bottom left plot of group (a 'group' is an analysis of identical markers).

☒ All plots are contour plots with outliers or pseudocolor plots.

☒ A numerical value for number of cells or percentage (with statistics) is provided.

Methodology

Sample preparation

For apoptosis assay, Mel624 melanoma cells were dissociated in 0.25% trypsin--EDTA (Gibco) at 37ºC for 5 min and the trypsin was inactivated with 10% FBS DMEM. Then the cells were was determined using the annexin V-FITC apoptosis detection kit (eBioscience, San Diego), according to the manufacturer’s instructions. Cell samples were then analyzed by BD FACSCalibur flow cytometer (BD Biosciences).

For Tumor infiltrating Lymphocytes analysis, Tumor tissue from B16F10 tumor-bearing mice (Day 14 after tumor cell inoculation)

was dissociated for 45min 37 by digestion with 2.5 mg/ml collagenase type IV (Worthington Biochemical, LS004188) and 100ug/ml DNAse (Sigma-Aldrich, DN25) in RPMI 1640 with 5% FBS. After digestion, tumor tissue was passed through 70-µm filters and mononuclear cells collected on the interface fraction between 40 and 80% Percoll. For intracellular staining, Tumor infiltrating lymphocytes from tumor tissues were stimulated with 50 ng/ml phorbol 12-myristate 13-acetate (Sigma-Aldrich, P8139) and 1ug/ml ionomycin (Fisher Scientific, BP25271) in the presence of Brefeldin A (BioLegend, 420601) for 4 h at 37°C. Cells were fixed and permeabilized using the BioLegend Kit (Catalog: 421002) for 30min at RT after surface staining.

Instrument

BD- FACSCalibur flow cytometer (BD Biosciences)
Fortessa 4-15 (BD Bioscience)

Software

For data collection and analysis, the software which is "BD CellQuest (TM)- Ver. 5.2" and "FlowJo software (version 10.5.3; FlowJo LLC)"

Cell population abundance

N/A

Gating strategy

Gating strategy was performed using positive and negative samples with single staining and combination. For Annexin V staining, positive Annexin V was defined for cells incubated with Annexin V-FITC. Negative Annexin V control was defined for cell non-incubated with Annexin V-FITC (non-stained). Same strategy is used for PI staining. Statistical analysis was performed on the double positive population.

Live cells (Zombie NIR negative) were gated using Zombie-violet (Catalog:423105) staining. FSC-A and FSC-A to exclude doublets. Lymphocytes were gated on SSC-A and FSC-A. CD4+ and CD8+ TILs gated on CD45+CD3+ cells. Gating strategies are shown in supplementary Figure S12A.

☒ Tick this box to confirm that a figure exemplifying the gating strategy is provided in the Supplementary Information.
